# Counterclockwise Virtual Reality–Based Embodiment of a Younger Self and Revisit of a Past Iconic Event in Older Adults: Between-Groups Study of Cognitive and Physical Performance

**DOI:** 10.2196/88338

**Published:** 2026-04-22

**Authors:** Domna Banakou, Reiya Itatani, Roger Montserrat, Clàudia Porta-Mas, David Bartrés-Faz, Ehud Bodner, Maria Mataro, Mel Slater

**Affiliations:** 1 Departament de Psicologia Clínica i Psicolbiologia, Universitat de Barcelona, Campus de Mundet Edifici CAVE Passeig de la Vall d'Hebron 171, Barcelona, Catalunya, 08035, Spain, 34 934039618; 2 Faculty of Medicine and Health Sciences, Universitat de Barcelona, Barcelona, Catalunya, Spain; 3 Interdisciplinary Department of Social Sciences, Bar-Ilan University, Ramat Gan, Israel

**Keywords:** counterclockwise, aging, virtual reality, reminiscence therapy, embodiment, body ownership

## Abstract

**Background:**

The original counterclockwise study carried out in the late 1970s provided an extreme example of “reminiscence therapy,” reporting improvements in older adults’ cognitive and physical functioning after they had lived for 5 days in a house set up as if decades earlier (the 1950s). We tested a virtual reality (VR) analog of this approach, enhanced by embodying participants in a virtual body that looked like themselves at the corresponding younger age.

**Objective:**

This study aimed to examine whether brief VR exposures combining (1) embodiment in a virtual body as one’s younger self and (2) immersion in an iconic past event improve age-related subjective and performance outcomes compared with a current-self VR control condition.

**Methods:**

We carried out a between-groups study with 23 healthy older adults (aged 65‐85 years; mean age 71.2, SD 4.03 years). Participants were randomly allocated to either a Young Self condition (n=11; mean 72.3, SD 4.17), where they were embodied in a virtual body that looked like themselves from the 1960s, or in a Current Self control condition (n=12; mean 70.1, SD 3.75), where participants were embodied in their current body. There were 5 sessions. In Session 1, participants completed a baseline assessment. There were then 2 VR exposures, approximately 1 week apart (Sessions 2‐3), and follow-ups at 1 week (Session 4) and approximately 2 weeks (Session 5) after the final VR exposure. Outcomes included subjective age, awareness of age-related change, World Health Organization–Five Well-Being Index, Trail Making Test performance, and physical functioning (eg, grip strength).

**Results:**

A hierarchical Bayesian analysis revealed that 1 week after the final VR exposure, those in the Young Self condition demonstrated lower subjective age than those in the Current Self condition (prob=.95). They had higher awareness of positive age-related change (prob=.89) and a higher score on the World Health Organization–Five Well-Being Index (prob=.84). Moreover, with respect to performance variables, they took less time to trace a trail (prob≥.99), made fewer mistakes in doing so (prob=.89), had greater right-hand (prob=.85) and left-hand (prob≥.99) grip strength. However, 2 weeks after their final VR exposure, these differences diminished apart from positive awareness of age-related change (prob=.82), trail-making mistakes (prob=.83), and left-hand grip strength (prob≥.99). Here, “prob” refers to posterior probability.

**Conclusions:**

The results demonstrate that even 2 short VR exposures, where people were embodied in their younger body and immersed in an iconic event from more than 50 years earlier, resulted in improvement in some age-related responses. This is encouraging for further research with more extensive VR experiences over a longer time period.

## Introduction

In the classic “Counterclockwise” study carried out in 1979, Langer [1] placed a group of 8 older men in a house for 5 days where everything was designed to be like the year 1959. The idea was that this backward time travel might lead to improvements in their mental and physical capabilities. The house was decorated to match the 1950s era, including furniture, types of lighting, and interior design. Newspapers and magazines from 1959 were also present. Even the television (TV) was from that time, playing black-and-white shows, and the radio played only 1959 programs, including the popular music of the time. The residents were encouraged to discuss issues current in 1959, such as the Cuban Revolution, the presidency of Dwight D Eisenhower, and popular sporting events, as if they were happening at that time. The residents brought photographs from the 1950s and were told to refer to themselves in those photos in the present tense. Moreover, they were required to wear 1950s-style clothing. There were no mirrors to show how they actually looked in the late 1970s, and no other modern objects were present. After the 5 days, the residents showed measurable improvements in physical strength, joint flexibility, posture, vision, hearing, and cognitive performance. Anecdotally, others judged them to look younger. When they first arrived at the house, the participants had trouble carrying their luggage; in contrast, when they finally left, they effortlessly carried their luggage to the waiting transport. Pagnini et al [2] carried out a quite faithful replication of the original study, but at the time of writing, the results did not appear to be available.

Counterclockwise was an early, intense, and immersive version of what today is referred to as reminiscence therapy (RT). RT involves encouraging older adults to recall and relive past experiences, often triggered by photographs and other objects from that time. It is often stated that it originated with Butler [3]; however, he discussed rather the “life review,” a condition where older adults reminisce (often quite negatively) about their past as death approaches, a behavior often viewed derogatorily by younger people and considered to have no beneficial effects. Butler did not explicitly propose this as a form of therapy, but rather described it as a phenomenon to be studied as something that occurs in later life and should be understood. He described it as a spontaneous phenomenon, possibly acting as an adaptive function for coping with old age. Nevertheless, these observations may be seen as foundational for the use of explicit reminiscence as a therapy. Indeed, Lewis and Butler [4] went on to develop “life review therapy” to enhance the spontaneous review to make it “more conscious, deliberate, and efficient.” Their method involved (1) requesting that the patient create autobiographies, (2) “pilgrimages” back to the places of birth and young adult life, (3) reunions with people from those times, (4) genealogical research to look into the lives of their forebears, (5) finding and looking at photo albums and scrapbooks, (6) summation of their life’s work, especially for those whose work was important to them, and (7) resurrecting their ethnic identity where this had been ignored or forgotten. It was suggested that the effectiveness of life review therapy depended on how well the process could help people resolve persistent feelings of resentment, guilt, bitterness, mistrust, and a sense of meaninglessness. As such, this type of life-review therapy was not aimed at improving cognitive or physical prowess, but rather to help people come to terms with the process of aging.

An early experimental study based on these ideas was carried out by Goldwasser et al [5], who treated older adults with dementia and compared group RT with supportive group therapy and a no-treatment group. The goal was to assess the effects on affective, cognitive, and behavioral functioning. The RT was carried out verbally by asking participants questions that evoked particular memories (eg, what they were doing during the war). They found positive effects on depression and affect in the RT group compared with the other 2 groups, but no other changes. Although the results were limited, the importance of this study was its formal between-groups experimental design and that it explicitly considered cognitive and behavioral functioning. While Goldwasser et al [5] (also refer to [6]) used verbal means to evoke memories, Yamagami et al [7] used “activity reminiscence therapy,” which requires participants to carry out activities in an old-fashioned way, such as cooking rice, including teaching it to younger adults or other members of the therapy group. They found some improvements in memory and indications of improvements in communication, interaction, and behavior 3 months posttreatment compared to 3 months pretreatment.

Such early examples of RT relied mainly on verbal recall of events through questioning and group discussion. However, studies such as [8,9] used photographs and other media. More recently, more technologically based methods have been used—for example, [10] discusses how to source items from the internet for personalized RT, and a systematic review can be found in [11]. Saragih et al [12] provide a meta-analysis of RT for people with dementia. Han et al [13] provide a systematic review and meta-analysis in general, concluding that RT has positive effects on cognition and that there is potential for further improvements, including the possible use of virtual reality (VR).

VR is increasingly used to deliver reminiscence-based interventions by immersing older adults in scenes from the past rather than relying solely on photographs or verbal recall. A recent review by Mao et al [14] concludes that VR-based RT is generally feasible and acceptable for older adults, with promising effects on emotional outcomes and some aspects of cognition, although heterogeneity in intervention content and outcome measures limits firm conclusions about effect size and durability. Randomized and longitudinal studies likewise suggest potential benefits for psychological well-being and cognitive functioning, sometimes exceeding those of traditional reminiscence approaches, but with mixed evidence for sustained cognitive change across follow-up [15]. In parallel, broader work on immersive VR with older adults emphasizes both the opportunity for ecologically rich interventions and the importance of designing for age-related sensory, motor, and novelty-related constraints [16].

In the outline of previous work above, we have seen that the approaches exposing participants to their past included talking, photographs, carrying out an activity, or being immersed in VR. These methods might be characterized as “exogenous” in the sense that the participant is always outside looking in or acting like something in the past. This leaves open a mechanistic question central to the counterclockwise premise: whether changing the self-perception of one’s age-related appearance, rather than only triggering autobiographical memory, is necessary to obtain measurable changes in age-related cognition and physical functioning. In this paper, we introduce a different paradigm, a VR version of counterclockwise. Many of the aspects of that original study were reproduced. The study was carried out in Barcelona, Spain, where participants were placed in a virtual room reminiscent of a living room in 1968, with decoration and furniture of that time. A black-and-white TV was playing the Eurovision Song Contest 1968, which took place at the Royal Albert Hall in London, where Massiel represented Spain and won the competition. This remains an iconic, highly memorable event for older adults today. Participants were then transported to the audience at the Royal Albert Hall, in a reproduction of the event, including the orchestra and backing singers, with the original soundtrack from the performance.

In VR, a person’s body can be visually substituted by a life-sized virtual body seen from a first-person perspective (1PP) [17,18]. Based on body tracking, the virtual body can be programmed to move synchronously with real body movements. When the body is seen from a 1PP and moves synchronously with actual body movements (visuomotor synchrony), evidence suggests that participants experience a strong illusion of “body ownership,” that is, the feeling that the virtual body is their body, even though they know that it is not. This was first shown by Petkova and Ehrsson [17], who used cameras mounted on top of a manikin looking down toward its body, and the video was streamed to a head-mounted display (HMD) worn by the participant. The manikin’s body and the corresponding areas of the real body were stroked by the experimenter, so that as the participant looked down, they would see the manikin’s body substituting their own, and the stroking on the manikin’s body would be felt synchronously on their real body. This led to body ownership over the manikin’s body, indicated by a questionnaire and a physiological arousal response when the manikin’s body was seen to be threatened by a knife. This method of inducing body ownership used visuotactile synchrony, inspired by the rubber hand illusion [19], rather than visuomotor synchrony, but the principle is the same—multisensory integration, including a 1PP view over the virtual body, leads to the body ownership illusion. Embodiment based on visuomotor synchrony has been demonstrated many times, for the hand [20,21] and for the full body [18,22-26], with some evidence that visuomotor may be more powerful than visuotactile [27].

Embodiment affords a fundamental difference from the original counterclockwise study: participants were embodied in a virtual body that looked like their younger selves from approximately that time. In the study reported in [1], the residents could not see themselves due to the absence of mirrors. In contrast, in our study, the participants were encouraged to spend some time looking at themselves in a virtual mirror and moving so that the mirror reflection moved synchronously and corresponded with their actual movements, thus supporting an illusion of ownership over their virtual body. We aimed to test whether a VR-based counterclockwise analog that combines (1) immersion in a salient autobiographical past context and (2) embodiment in a younger-looking virtual body produces measurable improvements relative to a current-self VR control condition. We hypothesized that participants who experienced their younger body and were transported to the immersive simulation of the Eurovision Song Contest 1968 performance by Massiel at the Royal Albert Hall would show greater improvement linked to shifts in subjective age and in measures of well-being, cognitive performance [28], and physical functioning than the control group, who were embodied in a virtual copy of their current body and transported to a recent performance of the same song by Massiel, as she looks today. Outcomes were assessed at baseline, after 2 VR exposures, and at follow-ups approximately 1 week and 2 weeks after the second exposure.

## Methods

### Experimental Design

**Figure 1.**
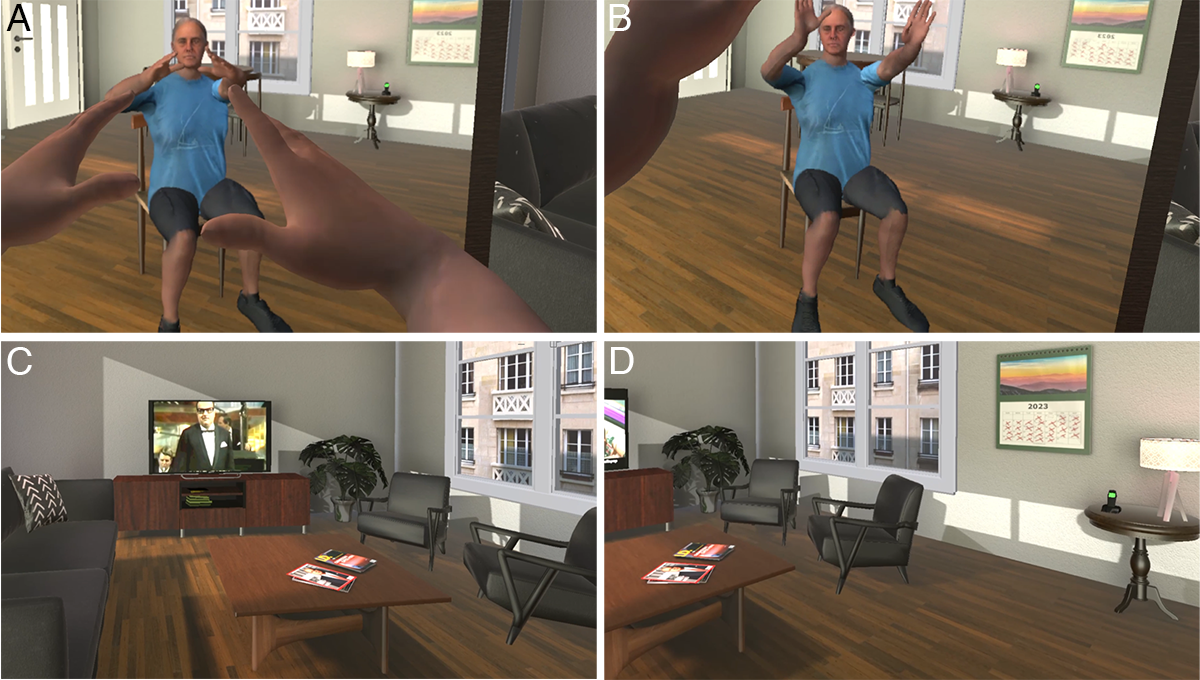
The Current Self virtual body and living room. (A and B) Participants see their virtual body by looking directly down at themselves and also in the mirror. As they move their upper body, the virtual body moves correspondingly and synchronously. (C) The living room view to the right of the participant, with the modern television (TV) showing the orchestra leader of the 1968 Massiel concert. (D) The room with the participant looking further to the right. The room is decorated in a modern style.

**Figure 2.**
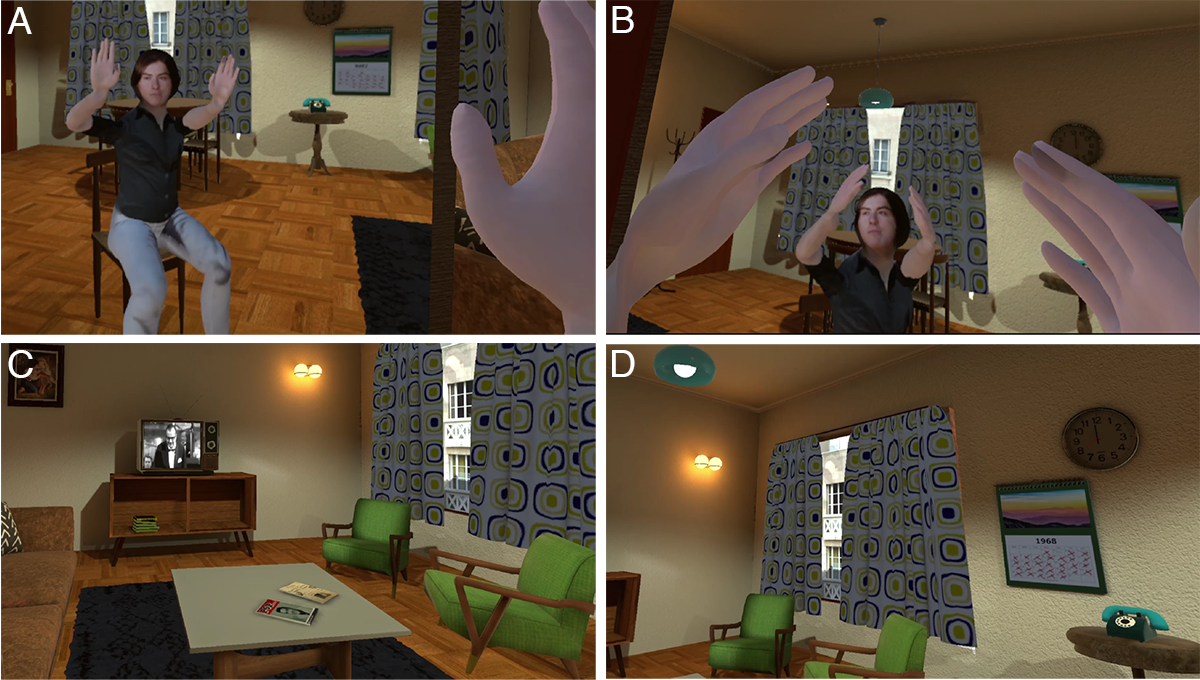
The Young Self virtual body and living room. (A-B) Participants see their young virtual body by looking down at themselves and also in the mirror. As they move their upper body, the virtual body moves correspondingly and synchronously. (C) The living room view to the right of the participant, with the monochrome television (TV) showing the orchestra leader of the 1968 Massiel concert. (D) The room with the participant looking further to the right. The room is decorated in a 1960s style.

**Figure 3.**
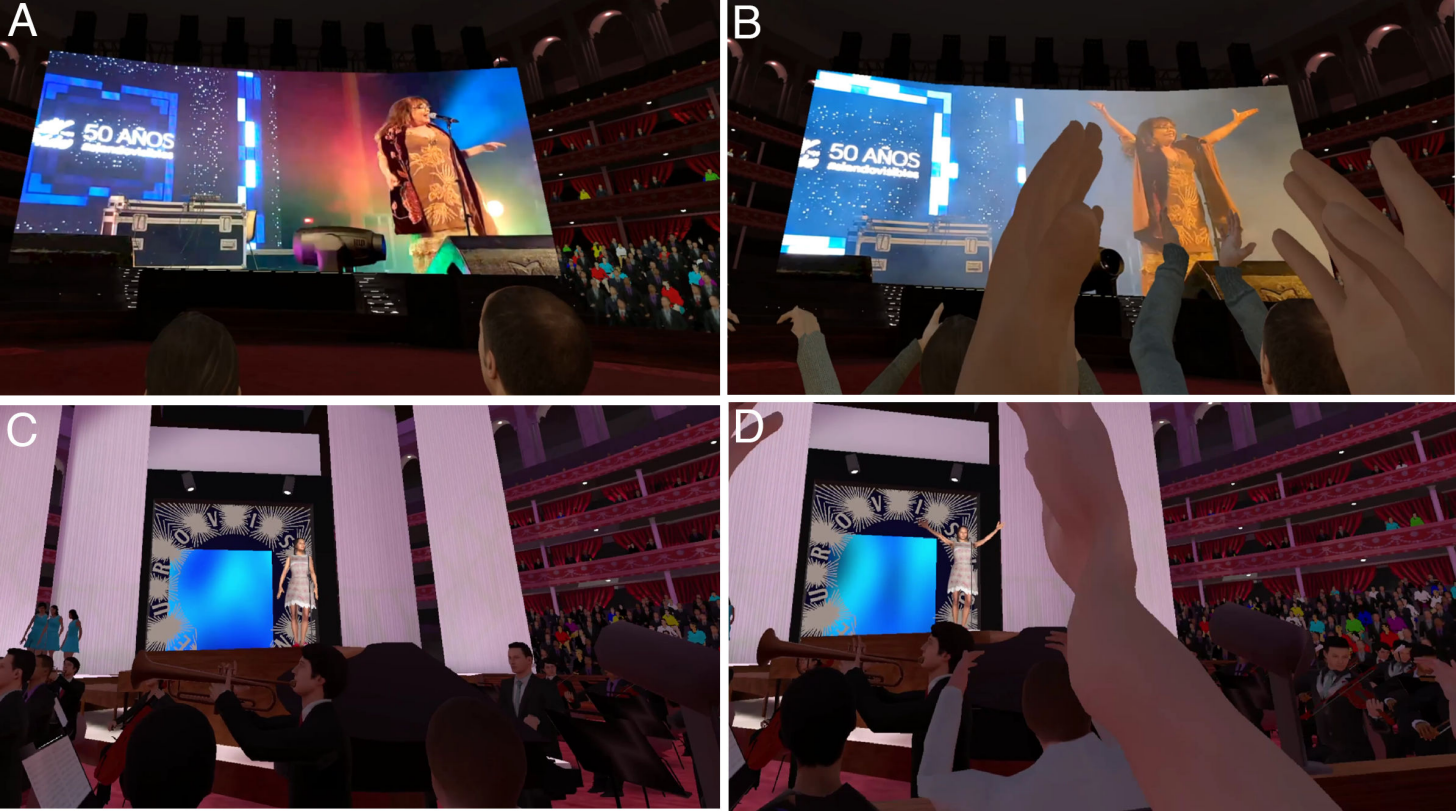
The concerts with the participant in the audience. (A) The 50th-anniversary concert shows the present-day appearance of Massiel on a large screen in a theater. (B) Toward the end of the performance of the song, the participant is clapping. (C) The 1968 performance at the Eurovision Song Contest, showing Massiel on stage, the backing singers, and the orchestra. (D) Toward the end of the concert, the participant is clapping.

The experiment followed a between-groups design with 1 factor with 2 levels: Current Self and Young Self. In the Current Self condition, participants were embodied in a virtual body that looked like their current appearance, in a modern living room (Figure 1). In the Young Self condition, participants saw themselves in their younger body in VR, located in a living room decorated as if it were in their youth (Figure 2). In each case, there was a TV in the room playing adverts and excerpts from programs of the time, followed by the start of a program about the Eurovision Song Contest 1968. In the Current Self condition, participants were then transported to a theater where they saw the present-day appearance of the singer Massiel performing the same song that won the Eurovision Contest in 1968 (“La la la”), celebrating the 50th anniversary, displayed in the VR environment on a large screen at the front of the theater (Figure 3A-3B). In the Young Self condition, participants were transported to a virtual Royal Albert Hall in 1968 and experienced attending the concert performance with the singer Massiel, her backing group, and the orchestra performing at the Eurovision Contest (Figure 3C-3D). In all cases, participants were embodied in their current or younger self virtual bodies from a 1PP (refer to Procedures). Hence, participants saw their virtual body both by looking down toward themselves directly and also as reflected in a virtual mirror. Participants’ real body movements were also mapped to their virtual bodies in real time using VR tracking devices. Participants were randomly allocated to either the Current Self or Young Self condition, and they completed 5 experimental sessions. Further details are given in the Procedures subheading and Table 1.

**Table 1.** Overview of the experimental sessions.

Session	Label	Timing	Description
1	Baseline	First visit	Consent + data protection forms; questionnaires; physical/performance measures: handgrip strength, walking speed, and balance.
2	VR^a^ Exposure 1	Approximately 1 week after Session 1 (on average)	VR: living room scene only; postsession questionnaires; handgrip strength, walking speed, and balance
3	VR Exposure 2	Approximately 1 week after Session 2 (on average)	VR: living room scene, then Royal Albert Hall with Massiel performing the winning song; postsession questionnaires; handgrip strength, walking speed, balance.
4	Follow-up 1	Approximately 1 week after Session 3	Questionnaires; handgrip strength, walking speed, and balance.
5	Follow-up 2	Approximately 2 weeks after Session 4	Questionnaires; handgrip strength, walking speed, and balance.

a VR: virtual reality.

### Participants

Thirty participants were recruited from the Department of Medicine (Faculty of Medicine and Health Sciences, University of Barcelona) among those attending cognitive assessments as part of a follow-up study on determinants of healthy aging. There were 7 dropouts: 5 for reasons unknown, 1 because of cardiology advice, and 1 due to the bereavement of a close family member. Finally, there were complete data on 23 participants (11 in the Young Self condition and 12 in the Current Self condition). The resulting distribution of participants by condition and sex is shown in Table 2. All participants were healthy Caucasian women and men aged 65-85 years and were not affected by any illness that prevented regular daily activity such as walking. All were retired.

Additional inclusion criteria included the following: no consumption of alcohol on the day of the experiment (before participation), no use of psychoactive medication, no existing conditions that caused dizziness, no strong susceptibility to motion sickness (eg, while traveling by car, boat, or airplane), no history of epilepsy, no cognitive impairment as assessed with the Mini-Mental State Examination (MMSE) [29-31], no major disabilities (such as requiring a wheelchair or assistive devices), no traumatic events during the age at which they would embody their younger self, and no history of neurological or major neuropsychiatric diagnoses (as determined by their physician). All participants were compensated for their participation with a payment of €100 (€20 for each session; conversion rate: approximately US $104). Multimedia Appendix 1 provides a summary of each demographic, background, and basic health variables and MMSE score for participants across conditions.

**Table 2.** Participant characteristics by condition, showing age and sex.

Condition	Male, n; mean (SD)	Female, n; mean (SD)	Total, n; mean (SD)
Current Self	4; 72.2 (4.19)	8; 68.9 (3.13)	12; 70.1 (3.75)
Young Self	6; 71.0 (4.15)	5; 73.8 (4.09)	11; 72.3 (4.17)
Total	10; 71.5 (3.98)	13; 70.9 (4.23)	23; 71.2 (4.03)

### Equipment

The study was carried out at Hospital Clinic, University of Barcelona. The VR experience was presented via the Meta Quest 2 HMD. The device has a display resolution of 1832×1920 pixels per eye and is powered by a Qualcomm Snapdragon XR2 processor with 6 GB of RAM, ensuring smooth performance. The headset displays at a 90-Hz refresh rate for fluid motion and reduced motion blur. It has built-in spatial audio and integrated tracking sensors. The HMD was attached to an MSI Katana laptop (Intel Core i7, RTX3060, and 16 GB of RAM) from which the program was executed. The Quest also has 2 handheld controllers. Both the head and hands are tracked in 6 degrees of freedom. Using this tracking, the motions of the upper virtual body could be matched to the movements of the real body using inverse kinematics, as described by Oliva et al [32].

### Procedures

Participants attended the 5 sessions described earlier in Table 1, with 2 VR exposures spaced approximately 1 week apart. Participants attended the experiment at prearranged times and were randomly allocated to the experimental or control condition. An overview of each session is described next, and a summary is given in Table 1.

#### Session 1: Baseline

Participants were provided with both verbal and written information about the experiment. They were required to complete and sign informed consent and data protection forms. Additionally, they were asked to complete a demographic questionnaire (ie, age, occupation, and so on) and a standardized screening questionnaire (MMSE) [29-31], to check cognitive functioning, as well as a single-item self-assessment of health question (“In general, how would you rate your health: excellent, very good, good, regular, or bad?”), which is a strong and consistent predictor of health outcomes.

They then completed a series of questionnaires that served as the pre-exposure assessments, including the Philadelphia Geriatric Center Morale Scale [33], the World Health Organization–Five Well-Being Index (WHO-5) [34,35], the Trail Making Test (TMT) [36,37], and underwent assessments for grip strength, walking speed [38], and balance [39] (refer to “Response Variables” subheading). All questionnaires were given on paper. Participants took short breaks between the various assessments or whenever requested. Before departing, a frontal photograph of the participant’s current appearance was taken to be used for the Current Self condition to produce their current body (Figures 1A and 1B), and they supplied a frontal photograph from their youth to be used for the Young Self condition (Figures 2A and 2B). The whole procedure lasted approximately 45 minutes.

#### Sessions 2 and 3: VR Exposure 1 to VR Exposure 2

Upon arriving at the laboratory for the first VR exposure, participants were introduced to the VR equipment, and the procedures were explained again. They were told that they could withdraw at any time without giving reasons and without any loss to themselves. Next, they were seated on a chair, put on the HMD, and were asked to hold the controllers. Participants remained seated throughout the VR exposure and were asked to only move their upper body, including their arms, hands, torso, and head, and to look around.

Once inside the virtual environment, participants found themselves seated in the virtual living room and were free to look around naturally. For the Current Self condition, the living room is shown in Figure 1C-1D, and for the Young Self condition in Figures 2C and 2D. A series of instructions was then given to them through audio guidance. Initially, participants were guided through a series of body movement exercises to explore the capabilities and real-time motion of their virtual bodies, including arm and head movements. Next, they were asked to look around in all directions and describe their surroundings. Following this orientation phase, they were instructed to focus on a TV located in the living room and observe its content. After a few advertisements, the TV started to show the Eurovision Song Contest 1968 performance by Massiel, after which the display gradually faded to black, marking the end of the VR exposure. Participants were then asked to complete the postexposure questionnaires and tasks. The session lasted approximately 45 minutes.

Approximately 1 week later, participants returned to the laboratory and were once again equipped with the VR headset and controllers. When the VR experience began, it started in the same living room as during the previous exposure. For those in the Current Self condition, where their virtual body was their current appearance, the TV showed a program about Massiel’s win more than 50 years earlier, and participants were transported to a modern theater where they watched a present-day performance of the same song by Massiel, shown on a large screen in front of the theater (Figures 3A and 3B). In other words, they saw Massiel as she looks today. For those in the Young Self condition, the TV program featured the opening of the Eurovision Song Contest 1968, announcing Spain as the winner, with Massiel’s performance of “La, la, la” (Figures 3C and 3D), as she looked at that time. As the scene transitioned, participants in the Young Self condition were transported to the audience at the Royal Albert Hall in London, where the event originally took place. Both groups experienced Massiel’s entire performance. The scenarios are provided in Movie S1 in Multimedia Appendix 2, and the concerts are illustrated in Figure 3. Participants were then asked to complete the postexposure questionnaires and tasks. The session lasted approximately 45 minutes.

#### Session 4: Follow-Up 1

Approximately 1 week after their second VR exposure, participants returned to the laboratory for the postintervention assessment (Session 4). They completed the grip strength, balance, and walking speed tasks, as well as the postexposure questionnaires. The session lasted approximately 30 minutes.

#### Session 5: Follow-Up 2

Approximately 2 weeks later, participants returned to the laboratory for the second postintervention assessment (Session 5) and repeated the tasks and questionnaires to assess changes over time. The session lasted approximately 30 minutes.

### Response Variables

#### VR-Related Variables

There were 2 types of response variables: the first type assessed the responses of participants to the VR experience. These are shown in Table 3 and include measures of presence, recognition of their virtual body, body ownership, and agency. We did not make inferences about these responses to the broader population; however, the extent of body ownership and agency was critical for the success of the method. Therefore, we present the results of these variables only descriptively.

**Table 3.** Response variables in relation to the virtual reality (VR) experience.

Variable	Interpretation
Presence and copresence	
there	Please rate your sense of being in the virtual environment on the following scale from 1 to 7, where 7 represents your normal experience of being in a place. Scale: 1 “Not at all” to 7 “Very Much”
real	To what extent were there times during the experience when the virtual environment was the reality for you? Scale: 1 “At no time” to 7 “Almost all the time”
visited	When you think back about your experience, do you think of the virtual environment more as images that you saw or more as somewhere that you visited? Scale: 1 “Images that I saw” to 7 “Somewhere that I visited”
beingin	During the time of the experience, which was strongest on the whole, your sense of being in the virtual environment or of being elsewhere? Scale: 1 “Being elsewhere” to 7 “Being in the virtual environment”
others	How much did you have a sense of being in the same space with other people? Scale: 1 “Not at all” to 7 “Very much so”
Recognition, body ownership, and agency	
recognized	How much did you recognize yourself when you saw your virtual body in the mirror? Scale: 1 “Not at all” to 7 “Very much so”
mybody	When you looked down toward yourself in the virtual reality, how much did the body you saw feel like your body (ignoring how much it looked like your body)? Scale: 1 “Not at all” to 7 “Very much so”
mirror	When you looked at yourself in the mirror, how much did the body you saw feel like your body (ignoring how much it looked like your body)? Scale: 1 “Not at all” to 7 “Very much so”
mymovements	How much were the movements of the virtual body your movements? Scale: 1 “Not at all” to 7 “Very much so”
Simulator sickness	
dizzy	How much dizziness or nausea did you feel while in the virtual reality? Scale: 1 “Not at all” to 7 “Very much so”

#### Age-Related Variables

The second type included age-related variables, comprising subjective measures (based on questionnaires) and performance-based measures, which assessed how a particular act—such as grip strength—was carried out. These are shown in Table 4.

The prespecified primary outcomes were subjective age (dlogage), WHO-5 well-being, TMT performance (Parts A and B: time and errors), and grip strength (left and right). Secondary outcomes included awareness of age-related change (AARC Positive and AARC Negative), morale (Philadelphia Morale Scale), balance, walking speed, and descriptive VR experience measures (presence, recognition, ownership, agency, and simulator sickness). Outcomes were assessed at baseline (Session 1), immediately following VR exposures (Sessions 2 and 3), and at follow-ups (Sessions 4 and 5).

**Table 4.** Response variables in relation to performance.

Variable	Interpretation	Rationale
Subjective variables		
subjectiveage	Participants were asked to assess their subjective age: “How old do you feel?” The response variable is measured as: dlogage=log(subjectiveage/age).	An indicator of subjective aging and aging identity that may be sensitive to embodiment. Beyond chronological age, it has been linked to important outcomes in older adulthood [28].
philadelphiatotal	Philadelphia Morale Scale—typically consists of 17 items, and the scoring range can vary depending on the format used. Each item is scored as either 0 (negative response) or 1 (positive response), making the total score range from 0 to 17.	Widely used measure of older adults’ morale/psychological well-being, capturing key dimensions such as agitation, attitude toward one’s own aging, and loneliness dissatisfaction [40], constructs that may plausibly shift with immersive or embodiment-related manipulations.
AARC^a^ Negative and AARC Positive	AARC^a^: items are scored on a 1 (strongly disagree) to 5 (strongly agree) Likert scale, where respondents rate their agreement or the extent to which they have experienced certain changes (eg, “With my increasing age, I realize that I have become wiser,” “With my increasing age, I realize that I am more prone to illness,” and so on); AARC^a^ Negative: higher scores mean the person perceives more negative changes with age. This may include a decline in health, memory, and physical ability, as well as social losses and reduced opportunities, and lower scores suggest fewer perceived downsides of aging; and AARC Positive: higher scores indicate that the person perceives more positive changes with age. Examples include increased wisdom, emotional regulation, deeper relationships, greater self-acceptance, or feeling more selective and purposeful in life. Lower scores suggest fewer perceived benefits of aging.	Widely used measure to understand how individuals perceive and react to the aging process, which can influence their overall well-being and behavior across both positive changes (eg, increased wisdom and improved emotional regulation) and negative changes (eg, physical decline and cognitive deterioration) [41]. It can help situate VR^b^ embodiment effects within a core aspect of aging identity, specifically how people notice and interpret age-related change.
wellbeingscale	WHO-5^c^ – scores / 100, where greater scores indicate greater well-being.	Brief measure of current subjective well-being in older adults, capturing positive mood and functioning over the past 2 weeks (eg, vitality, energy, relaxation, and interest in daily life). Because VR embodiment may influence affective state and self-related evaluations, the WHO-5 provides a concise, sensitive outcome for detecting short-term changes in overall emotional well-being [42].
Performance variables		
TMTAtime	Trail Making Test (TMT) Part A: participants connect a series of numbered circles (1-25) in ascending order as quickly as possible. This measures processing speed and visual-motor coordination. Time (in seconds) to complete the test.	Objective measure of visual scanning/visual search and processing speed with a clear visual–motor (psychomotor) tracking component, making it well-suited for quantifying performance in tasks that depend on fast visuomotor responding. It is widely used in aging research because it shows reliable age-related slowing and has normative data in older adult samples, supporting interpretation of performance differences in later life [43].
TMTBtime	TMT Part B: participants alternate between connecting numbered circles and lettered circles (eg, 1-A-2-B-3-C) in ascending order. This assesses cognitive flexibility, executive functioning, and the ability to switch between tasks. Time (in seconds) to complete the test.	Objective measure of visual scanning/visual search and processing speed with a clear visual–motor (psychomotor) tracking component, making it well-suited for quantifying performance in tasks that depend on fast visuomotor responding. It is widely used in aging research because it shows reliable age-related slowing and has normative data in older adult samples, supporting interpretation of performance differences in later life [43].
TMTAmistakes	TMT Part A: number of mistakes during the test (data for this measure were lost).	Objective measure of visual scanning/visual search and processing speed with a clear visual–motor (psychomotor) tracking component, making it well-suited for quantifying performance in tasks that depend on fast visuomotor responding. It is widely used in aging research because it shows reliable age-related slowing and has normative data in older adult samples, supporting interpretation of performance differences in later life [43].
TMTBmistakes	TMT Part B: number of mistakes during the test	Objective measure of visual scanning/visual search and processing speed with a clear visual–motor (psychomotor) tracking component, making it well-suited for quantifying performance in tasks that depend on fast visuomotor responding. It is widely used in aging research because it shows reliable age-related slowing and has normative data in older adult samples, supporting interpretation of performance differences in later life [43].
gripstrengthRmean	Grip Strength Test: right-hand force (kg), mean score.	Brief, objective indicator of overall muscle strength and physical capability in older adults. It is sensitive to age-related decline and is widely used as a marker of frailty/functional risk [44]. Lower grip strength is consistently linked to higher risk of disability and cardiovascular mortality in large-scale observational evidence and meta-analyses [45].
gripstrengthLmean	Grip Strength Test: left-hand force (kg), mean score; valid values were those >0 kg and <100 kg; and values were recorded as missing if the measurement differed by >20 kg for the same hand.	Brief, objective indicator of overall muscle strength and physical capability in older adults. It is sensitive to age-related decline and is widely used as a marker of frailty/functional risk [44]. Lower grip strength is consistently linked to higher risk of disability and cardiovascular mortality in large-scale observational evidence and meta-analyses [45].
balancemean	4-Stage Balance Test: mean score (in seconds); 0=lowest; 10=highest; and greater times are better (better balance).	Brief, objective measure of static postural control in older adults. Balance impairment is common with aging and is clinically important because poorer performance on this test is used in fall-risk screening and is associated with increased risk of future falls [46].
walktime	10-Meter Walk Test: comfortable walking time for a 10-meter distance (seconds).	Usual walking speed is a valid, reliable, and sensitive marker of functional status in older adults, and slower gait is associated with worse health outcomes [47]. It can be used to test the idea that if gait improves, embodiment transfers to functional motor behavior.

a AARC: awareness of age-related change.

b VR: virtual reality.

c WHO-5: World Health Organization–Five Well-Being Index.

### Implementation

#### Creating the Characters

The virtual bodies of the 3 backup singers, the conductor, and Massiel, the main singer, were created using the automatic 3D avatar generation system described in [48]. For the audience, high-resolution virtual humans were used within a 3.5-m radius from the participant, and these were programmed to look toward the participant at random intervals occasionally. Some of these characters were created using the same generation method [48], while others were adapted from the Microsoft Rocketbox Avatar Library [49]. For characters positioned between 3.5 m and 10 m away, an optimized per-joint impostor technique was applied to ensure rendering efficiency while maintaining visual fidelity [50]. Beyond 10 m, image-based crowd rendering was used to represent distant audience members [51]. In the Current Self condition, the audience consisted of virtual humans with an older appearance, whereas in the Young Self condition, the audience consisted of virtual humans with a youthful appearance. The animations of Massiel, the conductor, and the 3 backup singers were recorded using an OptiTrack motion capture system, and these recorded animations were applied to their corresponding avatars within the virtual scene.

#### Creating the Theaters

For the Young Self condition, the Royal Albert Hall was manually modeled in Blender (Blender Foundation) based on the official blueprints, comprising approximately 2900 seats, including 384 central audience seats and 48 orchestra seats. Each element, including stage layout, seating arrangement, and decorative structures, was modeled to match the proportions and architectural details of the original venue as accurately as possible.

#### Software and Implementation

These VR applications were developed using QuickVR [32]. QuickVR’s Avatar Tracking system synchronizes the movements of participants with their avatar using inverse kinematics to estimate and replicate the participant’s posture in real time. All 3D models, including the concert hall, stage, and environmental elements, were created using Blender. The overall application was implemented in Unity (Unity Technologies).

### Statistical Model and Analysis

Throughout, we used Bayesian statistical analysis, which is especially appropriate for the exploratory nature of this research. Rather than reaching conclusions (ie, via hypothesis testing), we present probabilities. A particular advantage is that we have many response variables, and with frequentist statistics, every additional statistical test further invalidates the meaning of the “significance level.” We would then have to resort to ad hoc methods such as Bonferroni corrections. In the Bayesian method, all response variables can be treated simultaneously in 1 overall model, and we can derive as many probability statements of interest as needed without impacting their validity. Moreover, unlike simply quoting results such as “*t*=3.2 and *P*<.05,” we explicitly state all assumptions and provide the source code online so that anyone can repeat the analysis, change prior distributions or the likelihood function, and examine the responses.

We report posterior means and 95% highest density intervals (HDIs), which can be interpreted as the range within which the true parameter value lies with 95% probability, given the data and model assumptions. In the Bayesian framework, before using the data, parameter values have prior probability distributions, reflecting the current state of knowledge. These distributions are updated by the data to yield the posterior distributions. The HDIs computed from the posterior distributions can be compared to the prior HDIs, and they typically focus on a much narrower range of values. Similarly, we can compute the posterior probability that a parameter is positive (or negative), indicating the probability that it is positively (or negatively) associated with the response variable.

We describe each component of the overall model together with the analysis of each response variable.

The linear predictor for any response variable refers to the right-hand side expression in the statistical model for that variable. For all of our response variables, the linear predictor is of the form:


individual+condition+session+(session×condition)

where individual represents the participant, condition is the main factor (Current Self=0 and Young Self=1), and session is (1,2,…,5). The last term is the interaction between condition and session.

The factors are represented by the following parameters:

$u_{j}$ is the random effect term of the jth participant (j=1,2,…,n=23)

$\alpha_{j}$ is the main effect of the jth Session (j=1,2,…,5)

$\beta_{k}$ is the effect of the kth condition k (k=1,2), where k=1,2 represent the Current Self and Young Self levels.

$\gamma_{jk},$j = 1,2; k = 1,2,…,5,is the interaction effect between session and condition, representing the additional effects of Young Self over the sessions.

For model identifiability, we set:


$$u_{1}=\alpha_{1}=\beta_{1}=\gamma_{1k}=\gamma_{j1}=0$$

j = 1,2; k = 1,2,…5

Hence, parameters are interpreted as offsets from the first participant (an arbitrary reference), the Current Self condition, and Session 1.

The statistical model is equivalent to a 2-way ANOVA model with random effects for participants. Here, the $u_{j}$ account for the effects of the individuals, and the $\alpha_{j},j=2,\ldots ,5$ account for the change from Session 1 to Sessions 2‐5 under the Current Self condition. The parameter $\beta_{2}$ reflects the difference between Young Self and Current Self at Session 1. The $\gamma_{jk}$ allow for interaction between the condition and session; that is, they provide the additional effect of Young Self compared with Current Self in Sessions 2‐5, beyond the effect given by $\beta_{2}$.

Hence, $\beta_{2}$ represents the initial difference between Young Self and Current Self in Session 1 (ie, before any intervention). The $\gamma_{2k},k=2,3,\ldots ,5$ represent the additional effects of being in the Young Self condition across sessions.

As explained at the start of this section, the Bayesian framework assigns prior probability distributions to the parameters, which are then updated by the data to produce posterior distributions:


posterior distribution ∝ likelihood × prior distribution

The likelihood is the distribution of the response variable conditional on the parameters.

Traditional ANOVA assumes a normal distribution for the likelihood. However, Figure S1 in Multimedia Appendix 3 shows the histograms of the subjective variables by condition (Current Self and Young Self), and Figure S2 likewise shows the distributions for the performance measures. In the case of the subjective variables, most of the distributions are skewed, and therefore, skew-normal distributions were used for the likelihoods. Skew-normal distributions have an additional parameter (in addition to the mean and SD) controlling skewness, and when this parameter is 0, the normal distribution is recovered. Therefore, in any case, the normal distribution is a special case of the skew-normal distribution. With respect to the performance variables, these are time or strength measures, which are nonnegative and typically follow a Gamma distribution. The alternative to using these likelihoods would be to attempt ad hoc transformations of the response variables to find one that transforms them to normality, which is, in general, not recommended [52]. We only used a log transformation log(subjectiveage/age) to ensure that this variable was based on a difference rather than a division, thereby reducing outliers and bringing its distribution into line with the other subjective variables. The full statistical models, including prior distributions, are given in Multimedia Appendix 4.

For analysis of the parameters, we concentrate on the HDIs of their posterior distributions. The HDI is analogous to a traditional CI, but it is an interval with a posterior probability. The 95% HDI is the smallest interval that contains 95% of the posterior distribution of the parameter. This is in contrast to a CI in frequentist statistics, which is such that if there were a large number of identical repetitions of the experiment, then 95% of the computed CIs would contain the true value of the parameter. However, any specific computed CI (ie, the one based on the actual available data) does not have 95% probability of containing the true value. In the frequentist interpretation, this specific computed interval either contains the population value of the parameter or does not; hence, only a probability of 0 or 1 could be assigned. Additionally, for each parameter, we provide the probability that it is positive or negative (ie, positively or negatively associated with the response variable).

**Figure 4.**
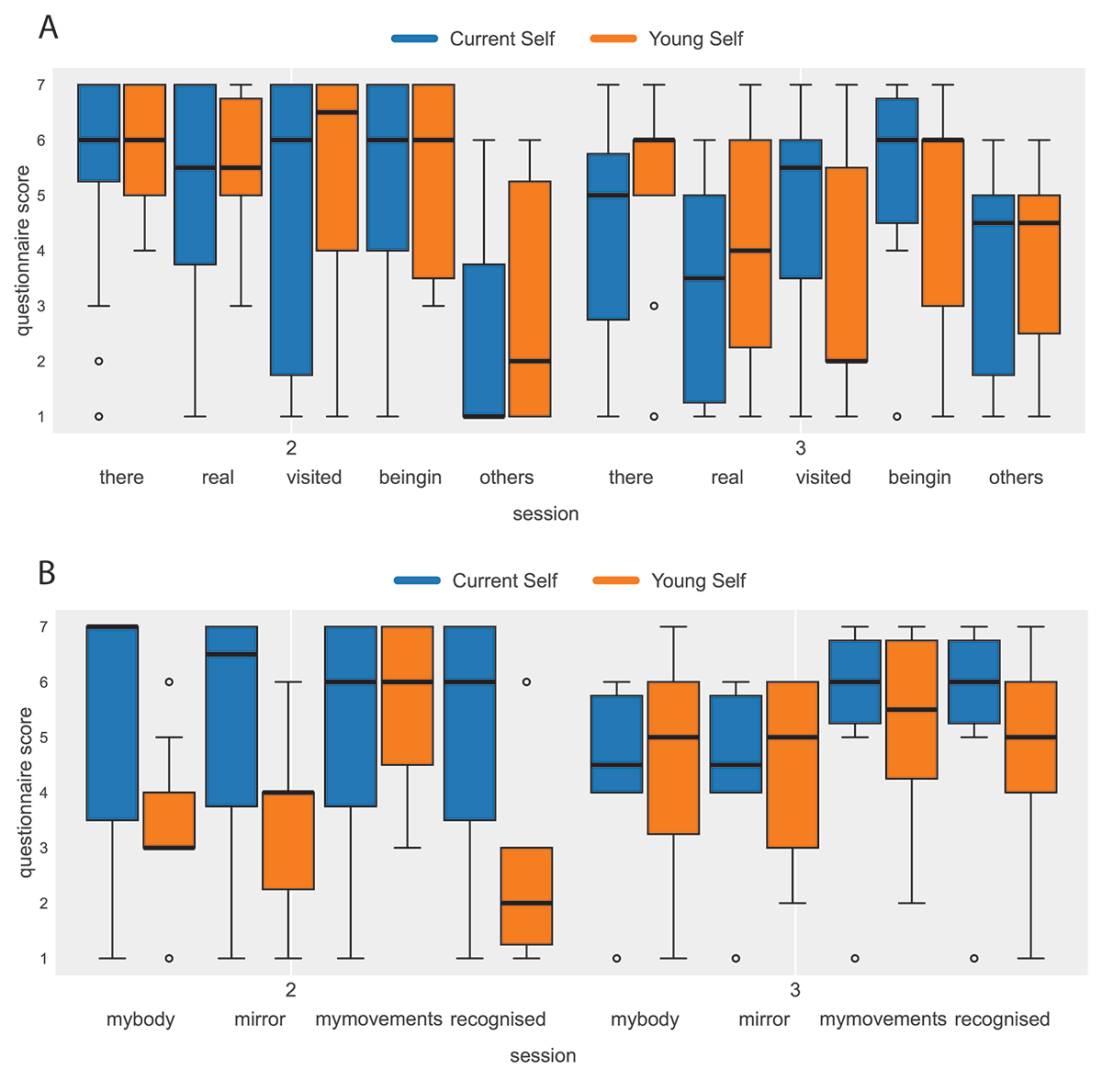
Box plots for presence- and body-related variables showing the medians and IQRs for variables listed in Table 3. (A) The variables related to presence, and (B) the virtual body–related variables.

All these results are provided in Tables S1 and S2 in Multimedia Appendix 5, which give summaries of the posterior distributions of all the parameters. Here, we present a graphical summary for each response variable, including box plots of the raw data by session and condition, and HDIs represented as horizontal line segments (Figures4  5). At the end of each line segment representing the HDI, the probability of the parameter being positive is shown.

**Figure 5.**
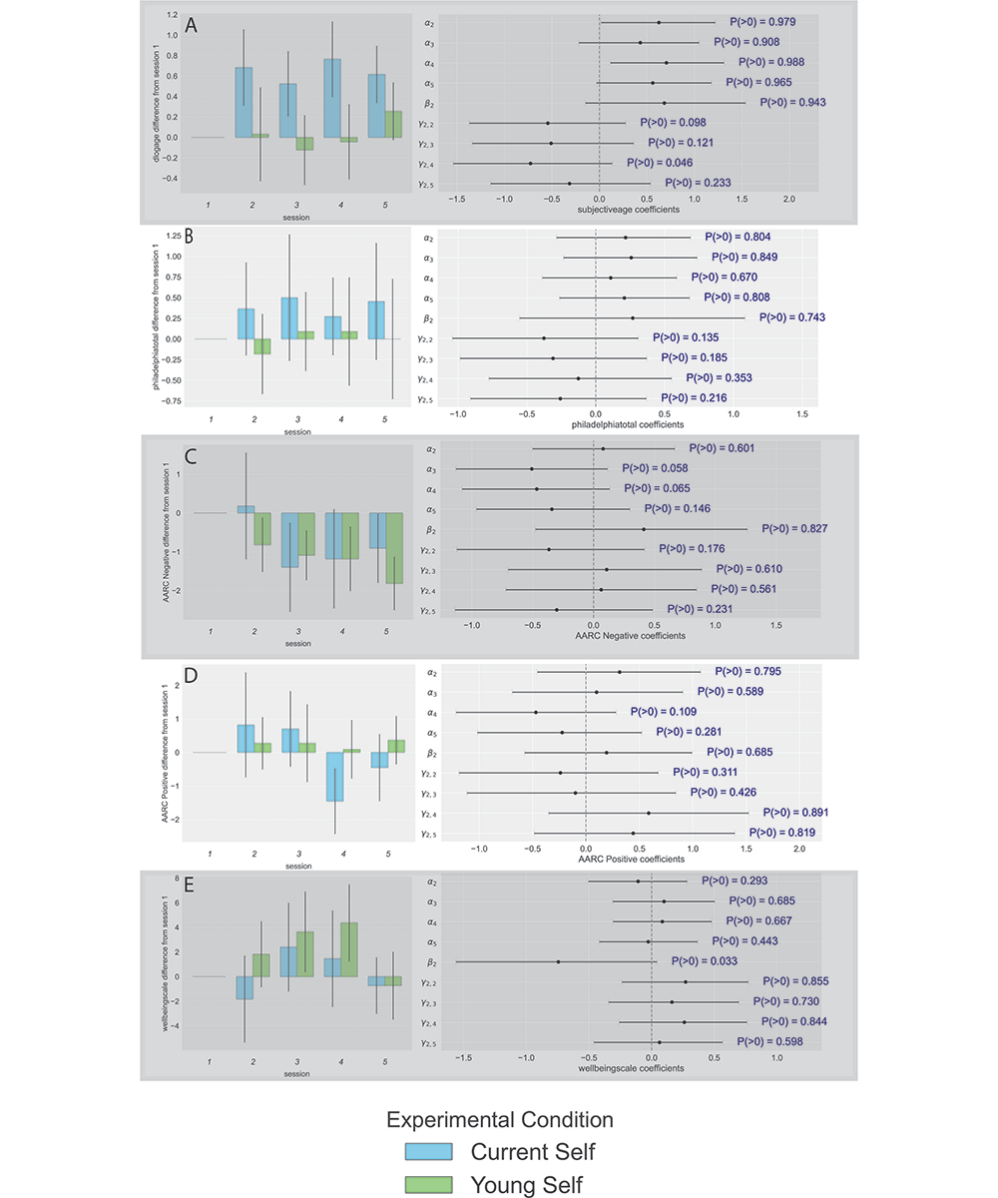
Bar charts of the subjective variables by session and condition (left) and 95% highest density intervals for the parameters of the model (right). (A) log(subjectiveage/age), (B) philadelphiatotal, (C) awareness of age-related change (AARC) Negative, (D) AARC Positive, and (E) wellbeingscale. *P*>0 is the probability that the corresponding parameter is positive.

### Ethical Considerations

The experiment was approved by the University of Barcelona Bioethics Commission (Comissió Bioètica of Universitat de Barcelona) and conducted in accordance with institutional ethical guidelines and international standards for the protection of human participants. Ethical measures included obtaining written informed consent, ensuring the right to withdraw at any point during or after the experiment, and maintaining confidentiality. Participants were informed that their data were strictly private and that the researchers would only access an anonymous ID number rather than any identifying information. After the final phase of the experiment, participants were debriefed about the purpose of the study. Because the intervention involved viewing and embodying a self-representation at a different life stage, participants were screened for factors that could increase distress (including major neuropsychiatric diagnoses and traumatic experiences during the target age period). Participants were reminded that they could pause or discontinue the VR exposure at any time without penalty.

## Results

### Baseline Characteristics

Table 5 summarizes participants’ demographic characteristics, background, and basic health variables, including ratings of subjective age and cognitive impairment. Overall, the groups were comparable, with no notable differences observed. Participants generally rated their health as good, and MMSE scores were above the standard 23/24 cutoff (overall mean 28.7, SD 0.78), indicating normal cognitive functioning [53].

**Table 5.** Summary of each demographic, background, and basic health variable.

Variable and meaning	Current	Young	Overall
Studies, n			
0=primary	3	—^a^	3
1=secondary	1	1	2
2=professional	2	4	6
3=university	5	6	11
StudyCompletionAge, mean (SD)			
At what age did you finish your studies?	19.8 (4.02)	21.4 (5.59)	20.6 (4.82)
Computing, median (IQR)			
Level of knowledge in information technologies (1=lowest and 7=highest)	5 (4-6)	4 (4-5)	4.5 (4-5)
Programming, median (IQR)			
Level of experience in programming (1=lowest [beginner] and 7=highest [expert])	1 (1-2)	1 (1-2)	1 (1-2)
VR^b^, median (IQR)			
How frequently has the participant had VR experiences (1=lowest [never] and 7=highest [a lot of times])	1 (1-5)	2 (1-5)	1.5 (1-5)
Gamesyear, median (IQR)			
How many times have you played video games during the last year? (0=0, 1=1‐5, 2=6‐10, 3=11‐15, 4=16‐20, 5=21‐25, and 6= ≥25)	0 (0-1)	0 (0-1)	0 (0-1)
Gamesweek, median (IQR)			
How many hours a week do you play video games? (0=0, 1=1, 2=2‐3, 3=3‐5, 4=5‐7, 5=7‐9, and 6= ≥9)	0 (0-0)	0 (0-0)	0 (0-0)
SubjectiveHealth, median (IQR)			
How would you rate your health in general? (0=excellent, 1=very good, 2=good, 3=regular, and 4=bad)	2 (1-2)	2 (2-2)	2 (2-2)
MMSETotal, mean (SD)			
Mini-Mental State Examination (total score; 0=lowest and 30=highest)	28.9 (0.70)	28.5 (0.82)	28.7 (0.78)

a Not available.

b VR: virtual reality.

### Presence and Embodiment

Figure 4A shows the box plots for the presence variables. There appears to be no difference in presence scores between the conditions, except for the variable “visited” in Session 3. In Session 2, the scores for “others” are low, whereas in Session 3, they are high. This is because there were no others in Session 2; thus, these data are consistent.

Figure 4B shows the results for the recognition, body ownership, and agency variables shown in Table 3. While in Session 2, people recognized their current selves, they did not recognize their younger selves. However, this was rectified in Session 3. For the Young Self condition, the body ownership scores for the variables mybody and mirror were low in Session 2, but this was rectified in Session 3. Agency-related scores (mymovements) were high in all conditions and sessions, reflecting the operation of the tracking system and the program.

This experiment was not about body ownership as such, and therefore, we do not make inferences about these variables to the broader population. Rather, we report them as manipulation checks. Participants’ self-reported ownership and agency ratings were generally consistent with the intended embodiment manipulation, particularly by the second VR exposure (Session 3), when recognition of the younger avatar improved.

### Simulator Sickness

The questionnaire included the following question: “How much dizziness or nausea did you feel while in the virtual reality?” on a 7-point scale ranging from 1 (“Not at all”) to 7 (“Very much so”). For both sessions and conditions, the median score was 1, with the IQR of 0. There were 2 outliers, a score of 7 in Session 2 (the first VR exposure) and a score of 3 in Session 3.

### Subjective Response Variables

#### Subjective Age

Here, we are interested in the difference between the person’s real age and their subjective age, that is, the age that they feel themselves to be. Figure 5A (left) shows the bar charts for the differences dlogage=log(subjectiveage/age), offset from Session 1 (ie, each value compared with the Session before any VR exposure). In each case, the mean dlogage in the Young Self condition is always lower than in the Current Self condition. However, the SEs are quite large. Figure 5A (right) shows the HDIs for each parameter in the statistical model fit for the response variable dlogage. The $\alpha_{j},j=2,...,5$ show that the Current Self results in high scores. Although the HDIs include 0, they are mostly to the right of the 0 line, and all the probabilities are greater than 0.908. The parameter $\beta_{2}$ again shows that at Session 1, the Current Self score is greatest (prob=.94). However, considering the $\gamma_{jk}$ in Session 2, the Young Self condition has a strong probability of subjective age being less than that of the Current Self condition (prob=1–.10=.99), a moderate probability at Session 3 (prob=1–.12=.88), and a strong probability in Session 4 (prob=1–.046=.954), but the advantage is lost by Session 5. Hence, overall, the subjective age from Sessions 2‐4 has a moderate to high probability of being less in the Young Self condition compared with the Current Self condition, but this effect is not maintained by Session 5.

#### Morale (Philadelphia Morale Scale)

In Figure 5B (left), we can see that the means are higher for the Current Self than for the Young Self condition, although the SEs are very high. Figure 5B (right) shows no effect of condition (Current Self vs Young Self) on this response variable. All the HDIs are wide and include 0, and none of the probabilities are salient.

#### AARC Negative

The bar charts for AARC Negative in Figure 5C (left) show that in both conditions, the negative evaluations decreased similarly over the sessions. The HDIs show more clearly that in the Current Self condition ($\alpha_{j}$) there is a decrease in the response in Sessions 3, 4, and 5, but that these are offset by moderate-probability increases in the corresponding Young Self condition. Overall, we conclude that there was no impact of the condition on AARC Negative.

#### AARC Positive

Figure 5D (left) suggests that in Sessions 2 and 3, there is little difference between the conditions. However, in Sessions 4 and 5, the Current Self means are lower than the Young Self means, although with large SEs. Figure 5D (right) suggests that the Young Self condition in Session 4 has greater scores than the Current Self condition (prob=.89) and similarly in Session 5 with a moderate probability of .82.

#### Well-Being (WHO-5)

Figure 5E (left) shows that the Young Self score is always at least as great as the Current Self score, with the most pronounced difference in Session 4. However, all the SEs are large. Figure 5E suggests that in Sessions 2 and 4, the Young Self scores are greater than the Current Self scores, with moderate probabilities of .86 and .84, respectively.

### Performance Variables

#### TMT Part A Completion Time

**Figure 6.**
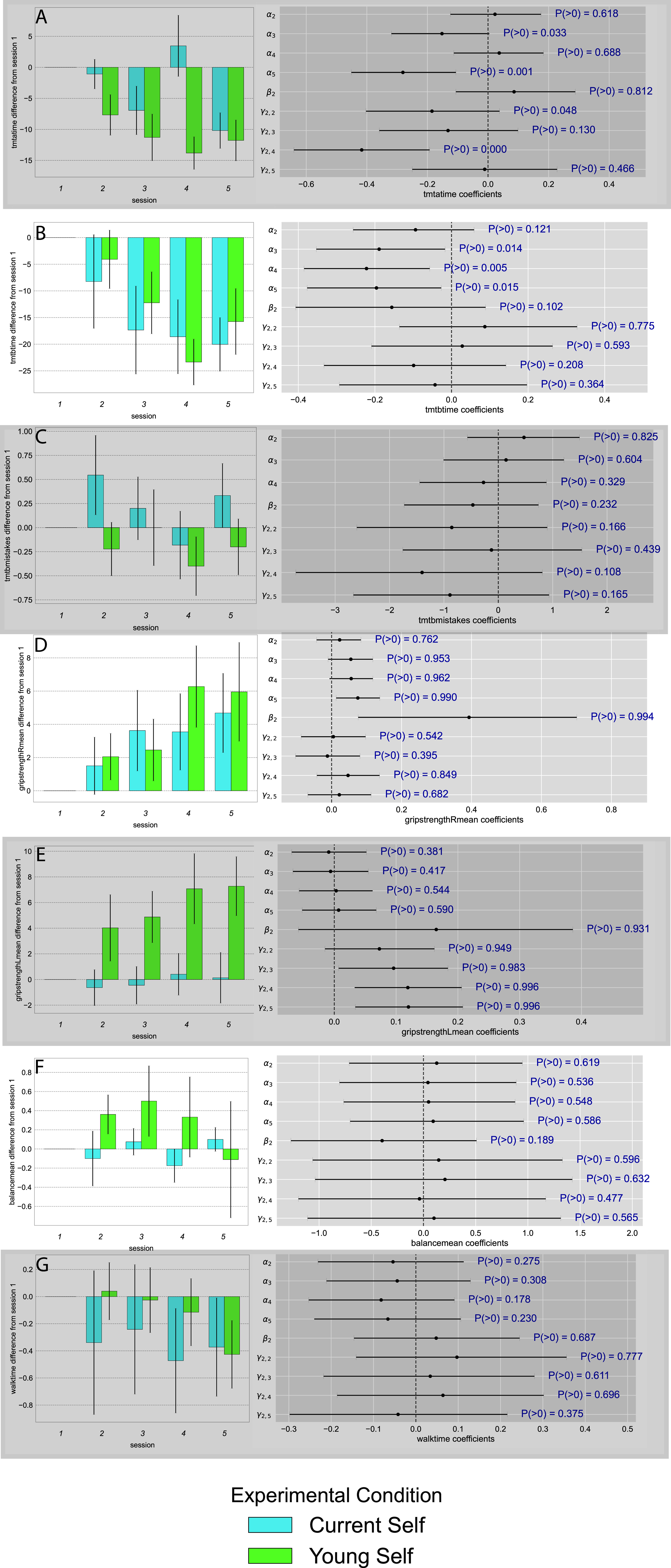
Bar charts of the performance variables by session and condition (left) and 95% highest density intervals for the parameters of the model (right). (A) tmtatime, (B) tmtbtime, (C) tmtbmistakes, (D) gripstrengthRmean, (E) gripstrengthLmean, (F) balancemean, and (G) walktime. *P(*>0) is the probability that the corresponding parameter is positive.

From Figure 6A (left), we can see that in both conditions, there is evidence of a reduction in completion time (tmtatime). In Session 2, the Young Self condition is lower than the Current Self (prob=1–.05=.95) and in Session 4 (prob≥.99; Figure 6A, right). However, by Session 5, there is no difference.

#### TMT Part B Completion Time

The bar charts in Figure 6B (left) do not show any marked difference between the conditions, and this is confirmed by the HDIs of the posterior distributions of the parameters (Figure 6B, right).

#### TMT Part B Errors

The bar charts in Figure 6C (left) indicate that the mean number of mistakes was always lower for the Young Self condition, and the HDIs and the probabilities moderately support this for Session 2 (prob=1–.17=.83), Session 4 (prob=1–.11=.89), and Session 5 (prob=1–.16=.84).

#### Grip Strength (Right Hand)

Figure 6D (left) shows a higher mean strength in the Young Self condition in Session 4, though with large SEs. This is moderately supported by the HDI and probability (prob=.85) for Session 4 (Figure 6D, right).

#### Grip Strength (Left Hand)

Figure 6E (left) shows a very clear advantage of the Young Self condition for all sessions, and this is backed up by the very high probabilities for the interaction terms (the minimum probability that Young Self is greater than Current Self is .95).

#### Balance (4-Stage Balance Test)

From Figure 6F (left), we can see that empirically, the balance mean score (in seconds; balancemean) is mostly greater for the Young Self condition; however, this is not supported by the statistical model (Figure 6F, right).

#### Gait Speed

Figure 6G (left) indicates that gait speed (walktime) is empirically greater for the Young Self condition for Sessions 2‐4, though with very large SEs. However, the statistical model shows no differences between the Current Self and Young Self conditions (Figure 6G, right).

### Summary

With moderate to high probabilities over the sessions, those in the Young Self condition reported lower subjective age than those in the Current Self condition. With moderate probabilities, the Young Self group had greater AARC Positive scores than the Current Self group in Sessions 4‐5. This means that they tended to perceive more positive changes with age. With moderate probabilities, the Young Self group had greater well-being scale scores than the Current Self group in Sessions 2 and 4. This scale indicates a greater sense of well-being. In Sessions 2 and 4, the Young Self group had lower TMT Part A times than the Current Self group, with high probability. This refers to the time required to complete the TMT. The number of mistakes made in the TMT Part B was lower in the Young Self than in the Current Self condition in Sessions 2 and 4, with moderate probabilities. The right-hand grip strength measured for the Young Self group was higher in Session 4 than that of the Current Self group, with moderate probability. The left-hand grip strength was greater in the Young Self group overall, with high probabilities.

The statistical analysis did not find any salient differences between the groups for Philadelphia total (a measure of morale), AARC Negative (reporting more negative changes with age), TMT Part B time (the time to complete a TMT), and balancemean (time standing on 1 leg).

It is interesting to see that differences between the 2 groups were most often found in Sessions 2 (the first VR session) and 4 (1 week after the VR exposures). However, it is important to note that all of the effects had vanished by Session 5, except for AARC Positive (with moderate probability), TMT Part B errors (with moderate probability), and left-hand grip strength (with high probabilities).

## Discussion

### Principal Findings

This study tested whether a VR analog of the counterclockwise paradigm, combining immersion in an autobiographical past context with embodiment in a younger-looking virtual body, produces measurable improvements in age-related subjective and performance outcomes relative to a Current Self VR control. We conducted a between-groups experiment in which a group of older adults was embodied in a virtual body that either resembled themselves as they are today (Current Self) or as they were in their teenage years or early 20s (Young Self). The Current Self group spent time in a modern virtual living room, where they saw their virtual body when looking down at themselves and when it was reflected in a mirror, and which moved synchronously with their own movements. They were then transported to the audience of a concert, where the modern-day Massiel performed the song that won her the Eurovision Song Contest in 1968. The Young Self group followed the same procedures, except they were embodied in their young body, in a 1960s living room, and were transported into the audience of a VR depiction of the original 1968 Massiel performance. Hence, they were either transported back in time with respect to their appearance and location (Young Self) or remained as they are now with respect to their appearance and the setting (Current Self). The experiment was conducted over 5 sessions (1: initial contact, 2: first VR exposure, 3: second VR exposure, 4: one week later, and 5: about two weeks later) with a number of subjective response measures and performance measures on each occasion.

Relative to the control, the Young Self condition showed the clearest evidence of benefit for subjective age and well-being, alongside improved TMT performance and higher grip strength, with effects most evident at Session 2 and at 1-week follow-up (Session 4). Several outcomes showed little evidence of between-group differences (eg, Philadelphia morale, AARC Negative, TMT Part B completion time, and balance), and most effects diminished by Session 5, but with persistent results observed for AARC Positive, TMT errors, and left-hand grip strength.

### Comparison With Prior Work

VR offers a potentially powerful method for delivering RT. Rather than looking at old photographs, newsreels, or home movies, participants can be directly immersed in scenes from the past. The systematic review of 22 studies by Ng et al [54] shows, most importantly, that across a wide range of interventions, there was little evidence of contraindications such as simulator sickness, and some positive outcomes for cognition. For example, Niki et al [55] carried out a study in Japan where VR was used among 10 participants aged at least 75 years who either saw scenes captured from a 360-degree camera (“live action”) or computer graphics–generated scenes themed from 1955 to 1980. They found that the method reduced anxiety and that there was some preference for the live action method, without contraindications such as simulator sickness. Likewise, a scoping review by Restout et al [56] found that participants positively appraised the use of 360-degree videos in this domain. Tominari et al [57] conducted a study in which 52 individuals with mild cognitive impairment participated in 8 weeks of RT, using either tablet-based panoramas (ie, nonimmersive) or conventional photos. Both groups demonstrated significant improvements in cognitive functions, with the tablet group showing higher morale. Similarly, Huang and Yang [58] carried out a pilot study involving 20 participants with dementia, where VR RT sessions—featuring scenes such as historical residences, music, and photographs—were held twice weekly for 3 months. While the VR intervention did not lead to significant cognitive changes immediately after the treatment, it did result in improvements in depressive symptoms, with evidence of decline over the next 6 months.

Khirallah Abd El Fatah et al [15] used personalized immersive VR reminiscence scenarios created using participants’ photo albums and videos, along with background information such as songs and friends gathered during interviews. These scenarios were compared with traditional RT and a control group that received standard care in older adult homes. While both the traditional and VR RT enhanced cognitive function and psychological well-being, the VR approach demonstrated greater efficacy.

While much RT research is mainly empirical, Lau et al [59] introduced a conceptual model (Immersion, Interaction, Imagination, and Impression) to help choose the content suitable for VR RT scenarios. This involved carrying out a survey conducted to identify suitable reminiscence materials for different age groups in Hong Kong, followed by the development of a prototype application. The prototype was then tested in a pilot study with occupational therapists, who provided positive feedback on its potential to offer a more engaging therapeutic experience compared to traditional methods. They noted that it helped elicit positive attitudes and motivation among older adults with dementia and improve their condition. The content was chosen to represent culturally significant scenes from various periods of Hong Kong’s past, tailored to specific age groups.

The most important aspect of our paradigm is that we do not use VR only to reproduce the appearance of the past, but we also change the body of the participant to represent the corresponding younger age. This was impossible in the original counterclockwise study, but it is straightforward in VR. As we have seen, other uses of VR in RT immerse people in representations of the past, but the issue of how they themselves appear is not considered. In this sense, we have two findings: (1) that RT in VR can produce several indicators of at least short-term improvement, and (2) the young virtual body can play an important role. It could be argued that our control condition should instead have been a condition without any virtual body self-representation. However, the fact that we found, on some variables, a difference between the Young and Current Self representations addresses this objection, since we have shown that changing the body has an effect. A study without any virtual body representation would not add additional information.

It might be thought that age may impact the propensity of older adults to achieve body ownership; in particular, they might not tolerate the small differences between proprioception and the seen movements of the virtual body that are inevitable in a VR setup. However, Martinelli et al [60] found that as people age and their proprioceptive sense declines, they rely more on the visual sense, so that movements of the virtual body would override such inaccuracies.

In relation to the impact of the representation of people at a different age, Reinhard et al [61] studied the effect on walking speed of embodying younger adults in older bodies and found that the speed after embodiment was lower. However, we did not find the converse effect, that embodying older adults in their younger body would increase their walking speed.

Our findings on subjective age and handgrip strength are also consistent with experimental and observational work on subjective age and physical functioning. In an experimental study, Stephan et al [62] provided older adults with positive, age-referenced feedback after an initial handgrip test; those who were led to believe that their performance was good for their age subsequently reported a younger subjective age and showed stronger handgrip on a second trial compared with controls who did not receive such feedback. In a complementary experiment, Swift et al [63] showed that framing a handgrip test as a comparison with younger adults (“Are they half as strong as they used to be?”) impaired older adults’ handgrip strength and persistence relative to a neutral comparison condition, indicating that age-related social comparison cues can immediately weaken physical performance. However, our results suggest that the Young Self condition functioned as a positive prime, successfully counteracting typical age-related decline by supporting the perceived physical capability of the participants through their rejuvenated virtual identity as their younger self and through exposure to an event from their youth.

Beyond these lab manipulations, Wang et al [64] found that community-dwelling older adults who felt younger than their chronological age demonstrated better performance on several physical fitness indicators, including grip strength. In a geriatric outpatient sample from Northern India, Bhattarai et al [65] reported that each additional kilogram of handgrip strength was associated with a higher likelihood of feeling younger than one’s peers. Large cross-sectional data from the Successful Aging Evaluation study similarly show that feeling younger than one’s chronological age is associated with better physical and mental health across the adult lifespan [66]. Taken together, this literature suggests that subjective age is modifiable and closely linked to markers of physical functioning. Against this backdrop, the present results—in which older adults were embodied in a younger virtual body—tended to reduce subjective age and enhance left-hand (and to some extent right-hand) grip strength relative to the Current Self condition and can be interpreted as a proof of concept for the current intervention. We suggest that VR-based reminiscence and embodiment paradigms tap into similar subjective-age mechanisms that have been demonstrated in non-VR experimental and epidemiological work.

Although not in the context of RT, there is related work in which people were embodied in bodies of different ages. For example, Hershfield et al [67] embodied younger adults in older bodies, and this impacted their behavior with respect to saving behavior. Tammy and Wu [68] embodied older adults (aged at least 60 years) who did not engage in vigorous activity in younger virtual bodies, finding that this led to greater perceived exercise exertion. Vahle and Tomasik [69] carried out studies with young and older adults to examine the effect of embodiment in differently aged bodies on cognitive and physical functioning. They found that younger participants embodying older avatars experienced performance declines in both cognitive and physical tasks compared with those with younger avatars. However, and in contrast to some of our findings, they did not find any significant performance differences in physical or cognitive domains for older participants embodying younger avatars vs older avatars.

### Limitations

The most important limitation of the study is the small sample size. This occurred for two reasons: (1) the difficulty in recruiting from this population, and (2) the relatively large drop-out rate due to illness or other factors more likely to occur among older participants. However, the Bayesian analysis is ideal for this. We start with prior distributions with high variance. Each additional data point updates the probability distributions of the parameters of the statistical model. Of course, the greater the number of participants, the narrower the posterior HDIs may become. If the data had no effect on the response variables, then the posterior distributions would not change much from the priors. However, in this experiment, if we compare the HDIs of the prior distributions (Table S2 in Multimedia Appendix 5) with the HDIs of the corresponding posterior distributions (Tables S1 and S2 in Multimedia Appendix 5; Figures5  6), we can see that they are much narrower than the priors. Hence, clearly, the data had an important effect.

Beyond sampling limitations, other procedural factors may have further constrained the study’s findings, and these should be addressed in future work. Due to technical limitations in the avatar reconstruction pipeline, the young avatars were often less realistic, having been derived from low-resolution, black-and-white historical photographs that remained suboptimal despite artificial intelligence (AI)–based enhancement. This discrepancy may have weakened participants’ sense of body ownership and inadvertently confounded condition assignment with avatar quality. AI-based enhancement may also produce unintended perceptual effects, such as eliciting an uncanny valley response [70]. Although this is unlikely in our manipulation, since the virtual body representations were still far from photorealistic, such effects may become more relevant as future enhancements increase realism. Future studies should therefore treat AI enhancement as a perceptual manipulation that requires validation, for example, by conducting brief pilot tests that include ratings of avatar eeriness and likability.

Additional problems included the need for experimenter-prompted verbal guidance during some VR sessions. Since the tasks were audio-guided, participants frequently required additional assistance, leading to interruptions that may have reduced presence for some participants. Future setups should incorporate in-scene virtual agents capable of offering guidance and companionship within the virtual environment, thereby preserving presence while minimizing experimenter intervention.

Hardware constraints may have also affected participant experience. For example, the headsets tethered to the computer limited mobility, and battery depletion or incompatibility with corrective glasses likely reduced comfort and visual fidelity, particularly among participants with pre-existing vision impairments. Furthermore, extending the duration of VR exposure would give participants more opportunity to acclimate to both the virtual environment and their embodied avatars, mitigating the disorienting effects of technological novelty in this age group. Finally, repeated administration of certain neuropsychological tests may have introduced practice effects or ceiling limitations, obscuring potential improvements. Future protocols should diversify cognitive measures to ensure sensitivity to genuine change.

Although the present experiment was not designed to investigate body ownership as such, the intervention necessarily relies on inducing a degree of body ownership in a self-resembling virtual body, which raises ethical and safety considerations. For example, experimental work suggests that immersive VR can transiently increase dissociative experiences, including depersonalization and derealization (DPDR) symptoms, particularly among individuals with higher preexisting dissociation tendencies [71,72]. However, recent cross-sectional survey findings indicate that most current VR experiences are unlikely to cause long-term DPDR symptoms for the majority of users [73]. In addition, avatar-based exposures can influence self-perception and body image attitudes [74]. A recent systematic review highlights how digital bodies might expose users to privacy and identity issues [75]. To mitigate potential risks, our study design incorporated several procedural safeguards, including rigorous participant screening for neuropsychiatric diagnoses and prior trauma, time-limited exposures, and a controlled laboratory environment. However, since we did not use standardized assessments for DPDR or body image changes, we cannot exclude the possibility of subtle or transient altered self-experiences. Future research should integrate validated state measures of dissociation, monitor for adverse psychological reactions both immediately postexposure and during follow-up, and establish prespecified stopping rules and support pathways for participants in distress.

Furthermore, although we manipulated embodied age between groups, our study does not directly test whether switching from a younger to an older or current virtual self (or vice versa) produces asymmetric emotional responses. However, participants in the Young Self condition necessarily transitioned from viewing and moving as a younger-looking self in VR to re-encountering their current real body when the headset was removed. This “re-entry” could plausibly produce a contrast effect, heightening AARC in a manner analogous to temporal self-comparison processes often implicated in nostalgia-related reactions [76]. However, since many of the responses (especially the reduction in subjective age) lasted at least 1 week after the VR exposure, this was unlikely to have been a problem. Future research should examine whether effects differ depending on the direction of embodied-age change and test “re-entry” procedures (eg, a brief grounding and debriefing phase or a gradual transition back to a current-self representation) designed to minimize potential negative contrast effects [77].

Overall, our experimental study has shown that a VR version of counterclockwise, where people are placed back in the past with a virtual body that looks like their younger self, can have a number of positive effects on responses that indicate self-improvement to overcome some effects of aging. However, most of these effects do not last beyond about 3 weeks. In a way, this is not surprising, because in the original counterclockwise experiment, the participants spent almost an entire week in the house decorated to look like the 1950s, in their 1950s clothes and with 1950s TV and magazines, whereas in our experiment, there were just 2 short exposures. However, our results hold promise for future work with multiple exposures, each with a different scenario.

Apart from the audience in the theater, the participants were alone in the VR scenarios. In earlier pilot studies, some participants suggested that it would have been useful if they had been accompanied by virtual human characters representing people from their past (in the Young Self) and present (in the Current Self) scenarios. This is possible, in that we would require photographs of others to construct their virtual bodies. However, for the Young Self condition, there may be ethical or data protection problems, for example, in representing people who were no longer alive and therefore unable to give permission, and for older adults, it would be quite likely that significant others from their youth (such as parents) would no longer be living, although some might be (siblings and friends). This may further complicate the process of recruitment, since not only would we need to find participants, but also those who had significant others able to permit the use of their photographs at a younger age. While this would make recruitment more difficult, it is likely to be worthwhile.

### Conclusions

More broadly, these findings suggest that VR-based interventions may be able to operationalize “age mindset” manipulations in a scalable and controllable way, allowing systematic testing of intervention “dose” (duration, repetition, and variety of scenarios) and mechanistic ingredients (past-context cues vs self-representation). If replicated in larger studies, this approach could provide a method for low-burden behavioral interventions targeting subjective age and related functional outcomes, with potential extensions to populations where reminiscence-based approaches are already used (eg, mild cognitive impairment or depressive symptoms), while maintaining careful attention to usability and safety in older adults.

## Supplementary material

10.2196/88338 Multimedia Appendix 1 Summary of each demographic, background, and basic health variable.

10.2196/88338 Multimedia Appendix 2 Video of the virtual reality scenario.

10.2196/88338 Multimedia Appendix 3 Histograms of the response variables.

10.2196/88338 Multimedia Appendix 4 The overall statistical model.

10.2196/88338 Multimedia Appendix 5 Summaries of the posterior distributions.

## Abbreviations

1PP: first-person perspective
AI: artificial intelligence
DPDR: depersonalization and derealization
HDI: highest density interval
HMD: head-mounted display
MMSE: Mini-Mental State Examination
RT: reminiscence therapy
TMT: Trail Making Test
TV: television
VR: virtual reality
WHO-5: World Health Organization–Five Well-Being Index

## References

[1] Langer EJ (2009). Counterclockwise: Mindful Health and the Power of Possibility.

[2] Pagnini F, Cavalera C, Volpato E (2019). Ageing as a mindset: a study protocol to rejuvenate older adults with a counterclockwise psychological intervention. BMJ Open.

[3] BUTLER RN (1963). The life review: an interpretation of reminiscence in the aged. Psychiatry (Abingdon).

[4] Lewis MI, Butler RN (1974). Life-review therapy. Putting memories to work in individual and group psychotherapy. Geriatrics.

[5] Goldwasser AN, Auerbach SM, Harkins SW (1987). Cognitive, affective, and behavioral effects of reminiscence group therapy on demented elderly. Int J Aging Hum Dev.

[6] Fry PS (1983). Structured and unstructured reminiscence training and depression among the elderly. Clin Gerontol.

[7] Yamagami T, Oosawa M, Ito S, Yamaguchi H (2007). Effect of activity reminiscence therapy as brain‐activating rehabilitation for elderly people with and without dementia. Psychogeriatrics.

[8] Baines S, Saxby P, Ehlert K (1987). Reality orientation and reminiscence therapy. A controlled cross-over study of elderly confused people. Br J Psychiatry.

[9] Davis RH (1981). Facilitating reminiscence with audiovisuals. Gerontologist.

[10] Chien HW, Liao SC, Huang SL, Chang CM, Chen HL, Chu HT (2016). Selecting internet videos and pictures for personalized reminiscence therapy. IJEIE.

[11] Lazar A, Thompson H, Demiris G (2014). A systematic review of the use of technology for reminiscence therapy. Health Educ Behav.

[12] Saragih ID, Tonapa SI, Yao CT, Saragih IS, Lee BO (2022). Effects of reminiscence therapy in people with dementia: a systematic review and meta-analysis. J Psychiatr Ment Health Nurs.

[13] Han Y, Zhu B, Huang D (2025). Efficacy of reminiscence therapy in improving cognitive decline: a systematic review and meta-analysis. Neurol Sci.

[14] Mao Q, Zhao Z, Yu L, Zhao Y, Wang H (2024). The effects of virtual reality-based reminiscence therapies for older adults with cognitive impairment: systematic review. J Med Internet Res.

[15] Khirallah Abd El Fatah N, Abdelwahab Khedr M, Alshammari M, Mabrouk Abdelaziz Elgarhy S (2024). Effect of immersive virtual reality reminiscence versus traditional reminiscence therapy on cognitive function and psychological well-being among older adults in assisted living facilities: a randomized controlled trial. Geriatr Nurs (Lond).

[16] Schaumburg M, Imtiaz A, Zhou R, Bernard M, Wolbers T, Segen V (2025). Immersive virtual reality for older adults: challenges and solutions in basic research and clinical applications. Ageing Res Rev.

[17] Petkova VI, Ehrsson HH (2008). If I were you: perceptual illusion of body swapping. PLoS ONE.

[18] Slater M, Spanlang B, Sanchez-Vives MV, Blanke O (2010). First person experience of body transfer in virtual reality. PLOS ONE.

[19] Botvinick M, Cohen J (1998). Rubber hands “feel” touch that eyes see. Nature New Biol.

[20] Kalckert A, Ehrsson HH (2012). Moving a rubber hand that feels like your own: a dissociation of ownership and agency. Front Hum Neurosci.

[21] Sanchez-Vives MV, Spanlang B, Frisoli A, Bergamasco M, Slater M (2010). Virtual hand illusion induced by visuomotor correlations. PLoS ONE.

[22] Peck TC, Seinfeld S, Aglioti SM, Slater M (2013). Putting yourself in the skin of a black avatar reduces implicit racial bias. Conscious Cogn.

[23] Banakou D, Groten R, Slater M (2013). Illusory ownership of a virtual child body causes overestimation of object sizes and implicit attitude changes. Proc Natl Acad Sci U S A.

[24] Banakou D, Slater M (2014). Body ownership causes illusory self-attribution of speaking and influences subsequent real speaking. Proc Natl Acad Sci U S A.

[25] Banakou D, Slater M (2017). Embodiment in a virtual body that speaks produces agency over the speaking but does not necessarily influence subsequent real speaking. Sci Rep.

[26] Banakou D, Beacco A, Neyret S, Blasco-Oliver M, Seinfeld S, Slater M (2020). Virtual body ownership and its consequences for implicit racial bias are dependent on social context. R Soc Open Sci.

[27] Kokkinara E, Slater M (2014). Measuring the effects through time of the influence of visuomotor and visuotactile synchronous stimulation on a virtual body ownership illusion. Perception.

[28] Westerhof G, Nehrkorn-Bailey A, Brothers A (2022). The effect of subjective aging on health and survival: a systematic review of longitudinal data. Innov Aging.

[29] Cockrell JR, Folstein MF, Copeland JRM, Abou-Saleh MT, Blazer DG (2002). Principles and Practice of Geriatric Psychiatry.

[30] Folstein MF, Robins LN, Helzer JE (1983). The Mini-Mental State Examination. Arch Gen Psychiatry.

[31] Kurlowicz L, Wallace M (1999). The Mini-Mental State Examination (MMSE). J Gerontol Nurs.

[32] Oliva R, Beacco A, Navarro X, Slater M (2022). QuickVR: a standard library for virtual embodiment in unity. Front Virtual Real.

[33] Lawton MP (1975). The Philadelphia Geriatric Center Morale Scale: a revision. J Gerontol.

[34] (1998). Wellbeing measures in primary health care/the DepCare Project: report on a WHO meeting: Stockholm, Sweden, 12–13 february 1998. https://iris.who.int/handle/10665/349766.

[35] Topp CW, Østergaard SD, Søndergaard S, Bech P (2015). The WHO-5 Well-Being Index: a systematic review of the literature. Psychother Psychosom.

[36] Vazzana R, Bandinelli S, Lauretani F (2010). Trail Making Test predicts physical impairment and mortality in older persons. J Am Geriatr Soc.

[37] Greenlief CL, Margolis RB, Erker GJ (1985). Application of the Trail Making Test in differentiating neuropsychological impairment of elderly persons. Percept Mot Skills.

[38] Bohannon RW (1997). Comfortable and maximum walking speed of adults aged 20-79 years: reference values and determinants. Age Ageing.

[39] Rossiter-Fornoff JE, Wolf SL, Wolfson LI, Buchner DM (1995). A cross-sectional validation study of the FICSIT common data base static balance measures. Frailty and Injuries: Cooperative Studies of Intervention Techniques. J Gerontol A Biol Sci Med Sci.

[40] Burton AE, Dean SE, Demeyin W, Reeves J (2021). Questionnaire measures of self-directed ageing stereotype in older adults: a systematic review of measurement properties. Eur J Ageing.

[41] Wettstein M, Kornadt AE, Wahl HW (2022). Awareness of age-related changes among middle-aged and older adults: longitudinal trajectories, and the role of age stereotypes and personality traits. Front Psychiatry.

[42] Li Y, Wilke C, Shiyanov I, Muschalla B (2024). Impact of virtual reality-based group activities on activity level and well-being among older adults in nursing homes: longitudinal exploratory study. JMIR Serious Games.

[43] Giovagnoli AR, Del Pesce M, Mascheroni S, Simoncelli M, Laiacona M, Capitani E (1996). Trail making test: normative values from 287 normal adult controls. Ital J Neurol Sci.

[44] Soysal P, Hurst C, Demurtas J (2021). Handgrip strength and health outcomes: umbrella review of systematic reviews with meta-analyses of observational studies. J Sport Health Sci.

[45] Gale CR, Martyn CN, Cooper C, Sayer AA (2007). Grip strength, body composition, and mortality. Int J Epidemiol.

[46] Salari N, Darvishi N, Ahmadipanah M, Shohaimi S, Mohammadi M (2022). Global prevalence of falls in the older adults: a comprehensive systematic review and meta-analysis. J Orthop Surg Res.

[47] Castell MV, Sánchez M, Julián R, Queipo R, Martín S, Otero Á (2013). Frailty prevalence and slow walking speed in persons age 65 and older: implications for primary care. BMC Fam Pract.

[48] Beacco A, Gallego J, Slater M Automatic 3D avatar generation from a single RBG frontal image.

[49] Gonzalez-Franco M, Ofek E, Pan Y (2020). The Rocketbox library and the utility of freely available rigged avatars. Front Virtual Real.

[50] Beacco A, Andujar C, Pelechano N, Spanlang B (2012). Efficient rendering of animated characters through optimized per‐joint impostors. Comput Animat Virtual Worlds.

[51] Tecchia F, Loscos C, Chrysanthou Y (2002). Image-based crowd rendering. IEEE Comput Grap Appl.

[52] Lo S, Andrews S (2015). To transform or not to transform: using generalized linear mixed models to analyse reaction time data. Front Psychol.

[53] Arevalo-Rodriguez I, Smailagic N, Roqué I Figuls M (2015). Mini-Mental State Examination (MMSE) for the detection of Alzheimer’s disease and other dementias in people with mild cognitive impairment (MCI). Cochrane Database Syst Rev.

[54] Ng WHD, Ang WHD, Fukahori H (2024). Virtual reality-based reminiscence therapy for older adults to improve psychological well-being and cognition: a systematic review. J Clin Nurs.

[55] Niki K, Yahara M, Inagaki M (2020). Immersive virtual reality reminiscence reduces anxiety in the oldest-old without causing serious side effects: a single-center, pilot, and randomized crossover study. Front Hum Neurosci.

[56] Restout J, Bernache-Assollant I, Morizio C (2023). Fully immersive virtual reality using 360° videos to manage well-being in older adults: a scoping review. J Am Med Dir Assoc.

[57] Tominari M, Uozumi R, Becker C, Kinoshita A (2021). Reminiscence therapy using virtual reality technology affects cognitive function and subjective well-being in older adults with dementia. Cogent Psychol.

[58] Huang LC, Yang YH (2022). The long-term effects of immersive virtual reality reminiscence in people with dementia: longitudinal observational study. JMIR Serious Games.

[59] Lau JSY, Tang YM, Gao G, Fong KNK, So BCL (2025). Development and usability testing of virtual reality (VR)-based reminiscence therapy for people with dementia. Inf Syst Front.

[60] Martinelli I, Risso G, Bertoni T (2025). From adolescence to old age: how sensory precision shapes body ownership during physiological aging. Front Hum Neurosci.

[61] Reinhard R, Shah KG, Faust-Christmann CA, Lachmann T (2020). Acting your avatar’s age: effects of virtual reality avatar embodiment on real life walking speed. Media Psychol.

[62] Stephan Y, Chalabaev A, Kotter-Grühn D, Jaconelli A (2013). “Feeling younger, being stronger”: an experimental study of subjective age and physical functioning among older adults. J Gerontol B Psychol Sci Soc Sci.

[63] Swift HJ, Lamont RA, Abrams D (2012). Are they half as strong as they used to be? An experiment testing whether age-related social comparisons impair older people’s hand grip strength and persistence. BMJ Open.

[64] Wang J, Yu J, Zhao X (2022). Is subjective age associated with physical fitness in community-dwelling older adults?. Int J Environ Res Public Health.

[65] Bhattarai U, Gautam A, Shrestha M (2024). Factors associated with subjective aging among older outpatients in Northern-India. J Frailty Sarcopenia Falls.

[66] Aftab A, Lam JA, Thomas ML, Daly R, Lee EE, Jeste DV (2022). Subjective age and its relationships with physical, mental, and cognitive functioning: a cross-sectional study of 1,004 community-dwelling adults across the lifespan. J Psychiatr Res.

[67] Hershfield HE, Goldstein DG, Sharpe WF (2011). Increasing saving behavior through age-progressed renderings of the future self. J Mark Res.

[68] Tammy Lin JH, Wu DY (2021). Exercising with embodied young avatars: how young vs. older avatars in virtual reality affect perceived exertion and physical activity among male and female elderly individuals. Front Psychol.

[69] Vahle NM, Tomasik MJ (2022). Younger and older adults’ cognitive and physical functioning in a virtual reality age manipulation. Front Aging.

[70] Tao Z, Liu Y, Qiu J, Li S (2025). Impact of virtual avatar appearance realism on perceptual interaction experience: a network meta-analysis. Front Psychol.

[71] Aardema F, O’Connor K, Côté S, Taillon A (2010). Virtual reality induces dissociation and lowers sense of presence in objective reality. Cyberpsychol Behav Soc Netw.

[72] Peckmann C, Kannen K, Pensel MC, Lux S, Philipsen A, Braun N (2022). Virtual reality induces symptoms of depersonalization and derealization: a longitudinal randomised control trial. Comput Human Behav.

[73] Barreda-Ángeles M, Hartmann T (2023). Experiences of depersonalization/derealization among users of virtual reality applications: a cross-sectional survey. Cyberpsychol Behav Soc Netw.

[74] Portingale J, Krug I, Liu H, Kiropoulos L, Butler D (2024). Your body, my experience: a systematic review of embodiment illusions as a function of and method to improve body image disturbance. Clin Psychol Sci Pract.

[75] Lin J, Latoschik ME (2022). Digital body, identity and privacy in social virtual reality: a systematic review. Front Virtual Real.

[76] Osborn H, Markman KD, Howell JL (2022). Nostalgia and temporal self-appraisal: divergent evaluations of past and present selves. Self Identity.

[77] Slater M, Gonzalez-Liencres C, Haggard P (2020). The ethics of realism in virtual and augmented reality. Front virtual real.

[78] Ageing study programs. Kaggle.

