# Supplementary material for: Counterclockwise Virtual Reality–Based Embodiment of a Younger Self and Revisit of a Past Iconic Event in Older Adults: Between-Groups Study of Cognitive and Physical Performance
Source: JMIR Form Res. 2026 Apr 22;10:e88338. doi: 10.2196/88338 (PMC13102333; doi:10.2196/88338)
Supplement: Multimedia Appendix 1 [file formative-v10-e88338-s001.docx]

**Table S1**. Summary of each demographic, background, and basic health variable

| **Variable** | **Meaning** | **Current** | **Young** | **Overall** |
| --- | --- | --- | --- | --- |
| Studies | 0 = primary  1 = secondary  2 =professional 3 = university | 3  1  2  5 | 1  4  6 | 3  2  6  11 |
| StudyCompletionAge | At what age did you finish your studies?  Mean ± SD | 19.8 ± 4.02 | 21.4 ± 5.59 | 20.6 ± 4.82 |
| Computing | Level of knowledge in Information Technologies  1 = lowest  7 = highest  Median (IQR) | 5 (2) | 4 (1) | 4.5 (1) |
| programming | Level of experience in programming  1 = lowest (beginner)  7 = highest (expert) | 1 (1) | 1 (1) | 1 (1) |
| VR | How frequently has the participant had VR experiences  1 = lowest (never)  7 = highest (a lot of times) | 1 (4) | 2 (4) | 1.5 (4) |
| Gamesyear | How many times have you played videogames during last year?  0 = 0  1 = 1-5  2 = 6-10  3 = 11-15  4 = 16-20  5 = 21-25  6 = >25 | 0 (1) | 0 (1) | 0 (1) |
| Gamesweek | How many hours a week do you play videogames?  0 = 0  1 = 1  2 = 2-3  3 = 3-5  4 = 5-7  5 = 7-9  6 = >9 | 0 (0) | 0 (0) | 0 (0) |
| SubjectiveHealth | How would you rate your health in general?  0 = excellent  1 = very good  2 = good  3 = regular  4 = bad | 2 (1) | 2 (0) | 2 (0) |
| MMSETotal | Mini-Mental State Examination - Total Score  0 = lowest  30 = highest | 28.9 ± 0.70 | 28.5 ± 0.82 | 28.7 ± 0.78 |
