# Supplementary material for: Counterclockwise Virtual Reality–Based Embodiment of a Younger Self and Revisit of a Past Iconic Event in Older Adults: Between-Groups Study of Cognitive and Physical Performance
Source: JMIR Form Res. 2026 Apr 22;10:e88338. doi: 10.2196/88338 (PMC13102333; doi:10.2196/88338)

**Figure S1**. Histograms by condition of each of the subjective variables from Table 3. (A) log(subjectiveage/age) standardized to variance 1, (B) philadelphia total, (C) AARC Negative, (D) AARC Positive, (E) wellbeingscale, and (B-E) are standardized to mean 0 and variance 1.


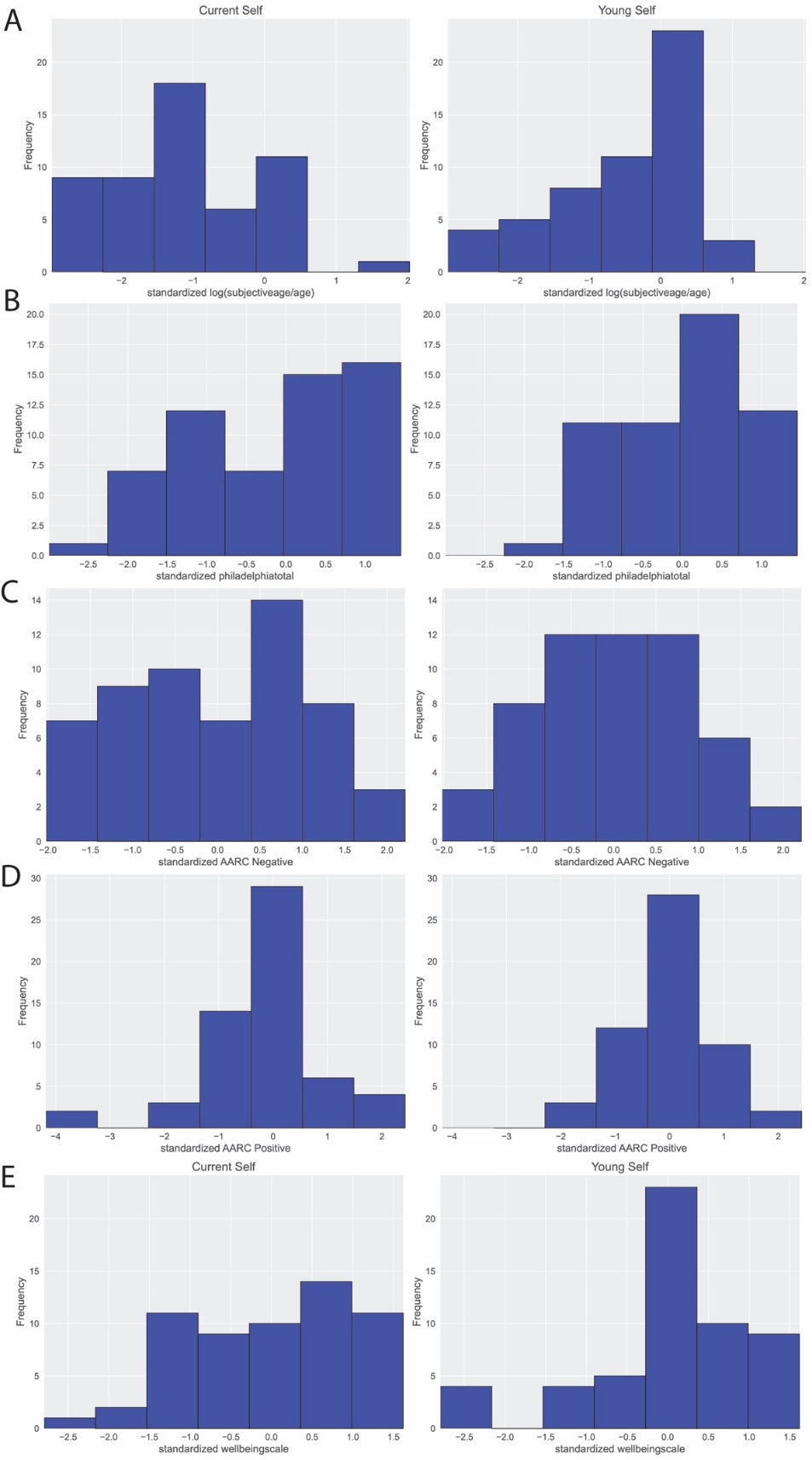


**Figure S2**. Histograms by condition of each of the performance variables from Table 3. (A) tmtatime, (B) tmtbtime, (C) tmtbmistakes, (D) gripstrengthRmean, (E) gripstrengthLmean, (F) balancemean, and (G) walktime.


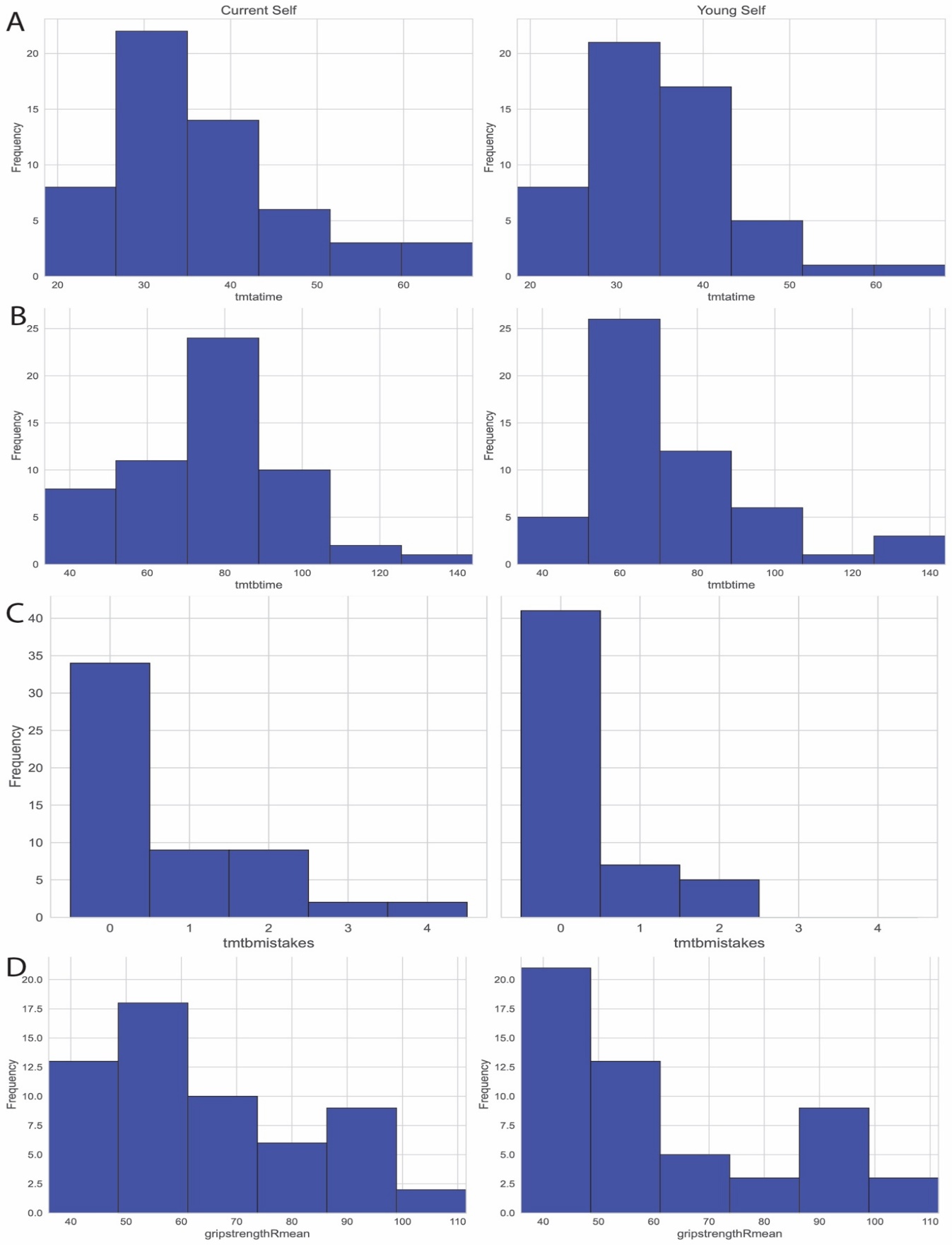


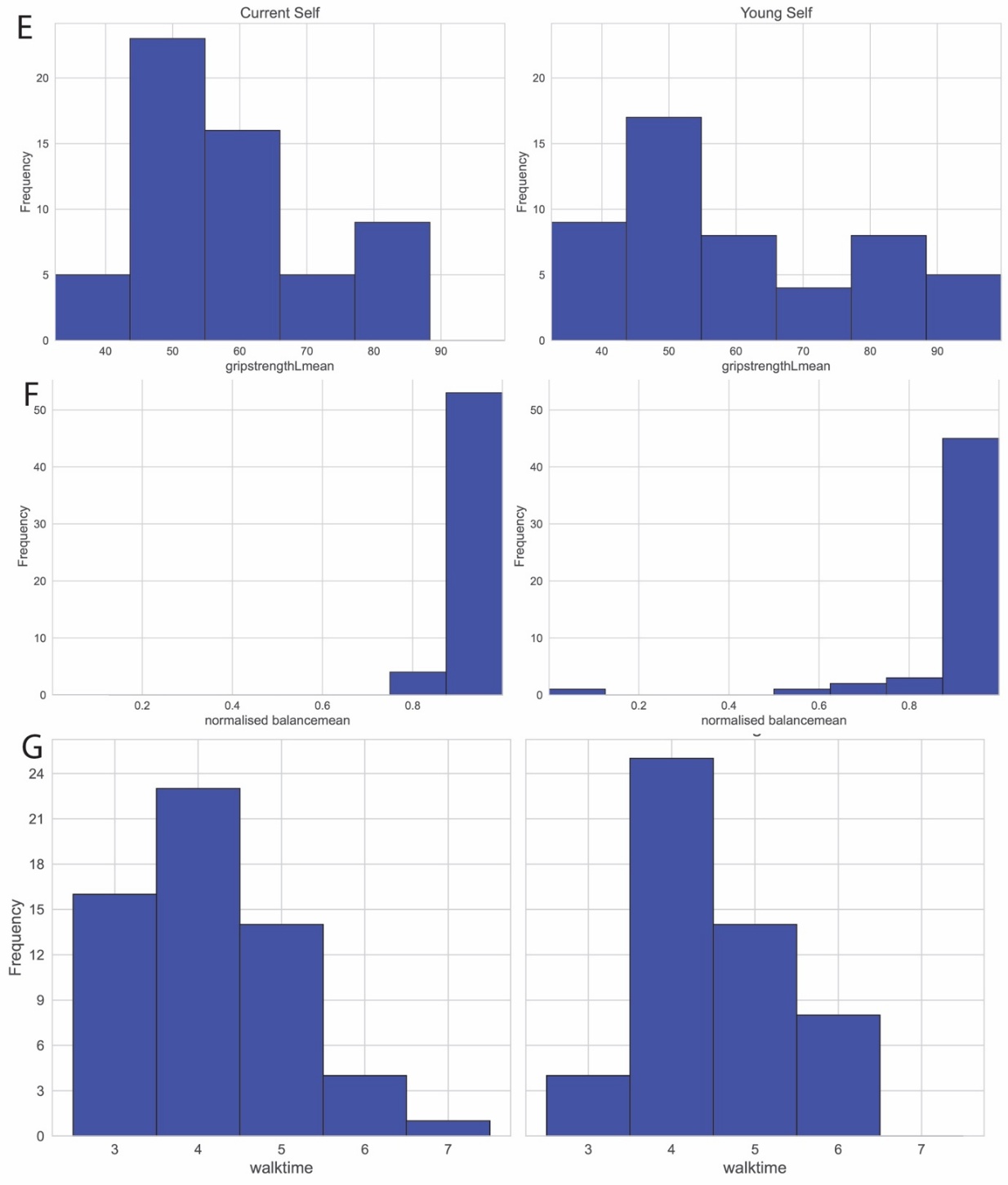

Supplement: Multimedia Appendix 3 [file formative-v10-e88338-s003.docx]
