# Supplementary material for: Counterclockwise Virtual Reality–Based Embodiment of a Younger Self and Revisit of a Past Iconic Event in Older Adults: Between-Groups Study of Cognitive and Physical Performance
Source: JMIR Form Res. 2026 Apr 22;10:e88338. doi: 10.2196/88338 (PMC13102333; doi:10.2196/88338)
Supplement: Multimedia Appendix 4 [file formative-v10-e88338-s004.docx]

## The Overall Statistical Model

The linear predictor for each response variable has the following form:

$$\eta_{i}=\mu+u_{ind\left[ i \right]}+\alpha_{sess\left[ i \right]}+\beta_{C\left[ i \right]+1}+\gamma_{C\left[ i \right]+1,sess\left[ i \right]}$$

$$i=1,2,\ldots,N=115$$

(1)

where $\mu$ is the general mean.

$ind=\left[ 1,1,1,1,1,2,2,2,2,2,\ldots,n,n,n,n,n \right]$ where $n=23$, the number of individuals, and $N = 115$ is the length of $ind$.

$u_{j}$ is the effect of the $j$th participant ($j = 1,2,\ldots,n=23$)

$$C\left[ i \right]=\left\{ \begin{matrix} 0, Current body \\ 1, Young body \end{matrix} \right.$$

$$i = 1,2,\ldots,N$$

Hence this has the form:

$C=\left[ 0,0,0,0,0,1,1,1,1,1,\ldots.,0,0,0,0,0 \right]$ representing the experimental condition of each participant.

$\alpha_{j}$ is the effect of the $j$th session ($j = 1,2,\ldots,5$), where

$sess=\left[ 1,2,3,4,5,1,2,3,4,5,\ldots,1,2,3,4,5 \right]$ represents the sequence of the sessions.

$\beta_{k}$ is the effect of the $k$th condition $k$ ($k = 1,2$), where $k=1,2$ represent the Current Self and Young Self levels.

$\gamma_{jk}$ where $j = 1,2; k = 1,2,\ldots,5$, is the interaction effect between session and condition.

Here, the $u_{j}$ account for the effects of the individuals, the $\alpha_{j}$ account for the effect of the sessions, $\beta_{i}$ account for the conditions, and the $\gamma_{jk}$ allow for interaction between the conditions and sessions.

For identifiability we must set:

$$u_{1}=\alpha_{1}=\beta_{1}= \gamma_{1k}=\gamma_{j1}=0$$

$$j = 1,2; k = 1,2,\ldots5$$

Hence parameters are interpreted as *offsets* from the first participant (this is arbitrary and unimportant), the Current Self and Session 1.

We fit this model twice, once for all of the subjective variables and then again, separately for all the performance variables. The reason was to avoid too many response variables in one overall model.

## Subjective Response Variables

All response variables were standardised to have mean 0 and standard deviation 1, except with a modification for subjective age. Figure S1(A) of Multimedia Appendix 1 shows the distribution of $log\left( subjectiveage/age \right)$, standardized to variance 1. It was not standardized to have mean 0, since the original 0 is important, indicating where subjectiveage and age are the same.

Multimedia Appendix 1, Figure S1 shows the histograms of the response variables, and it can be seen that almost all are skewed, some right-skewed, some left-skewed, and some possibly not. Hence we used the skew normal distribution throughout [1,2].

The skew normal distribution has 3 parameters: $\xi$ which is location, $\sigma>0$ which is scale, and $\kappa$ which is shape. If $\kappa=0$ then the result is a normal distribution, if $\kappa>0$ then it is right-skewed, and otherwise it is left-skewed.

The mean of the skew normal distribution in terms of these parameters is:

$$mean = \xi+\sigma\delta\sqrt{\frac{2}{\pi}}$$

with

$$\delta=\frac{\kappa}{1+\kappa^{2}}$$

Hence if we make the location parameter as

$$\xi=\eta- \sigma\delta\sqrt{\frac{2}{\pi}}$$

where $\eta$ is the linear predictor from Eq (1), then the mean of the distribution will be $\eta$.

The priors for all response variables are shown in Table S1.

**Table S1**. Prior distributions for the parameters common to all response variables, with $\sigma=2.5$.

| **Parameter** | **Meaning** | **Prior** |
| --- | --- | --- |
| $\mu$ | Grand mean | $normal\left( 0,\sigma\right)$ |
| $u_{j}$, $j = 2,3,\ldots,n=23$ | Participant effect | $normal\left( 0,1 \right)$ |
| $\alpha_{j}, j=2,\ldots,5$ | Session effect | $normal\left( 0,\sigma\right)$ |
| $\beta_{i}, i=2$ | Condition effect | $normal\left( 0,\sigma\right)$ |
| $\gamma_{jk},j=2;k=2,3,\ldots,5$ | Interaction between session and condition | $normal\left( 0,\sigma\right)$ |
| $\sigma$ | Scale | $normal\left( 0,\sigma\right)$truncated at lower bound 0. |
| $\kappa$ | Shape | $normal\left( 0,\sigma\right)$ |

## Performance Response Variables

The linear predictor is shown in Eq. (1). However, the response variables could not be treated all in the same way because of their individual characteristics. We consider them next.

### Time and Strength Variables

The variables TMTAtime and TMTBtime, gripstrengthRmean, gripstrengthLmean, walktime are all waiting time variables or the amount of force registered on a scale. Such variables, with lower bounds at 0, typically follow a Gamma distribution, especially the time ones, and this is backed up by the histograms shown in Multimedia Appendix 1, Figure S2. The Gamma distribution has two parameters shape ($\kappa>0)$ and rate ($\lambda>0)$, and the mean of the distribution is $\kappa/\lambda$. Hence for the likelihood for any of these response variables $y$, we use the distribution:

$$y\sim Gamma\left( \mu\phi,\phi\right)$$

where $\phi>0$ is the rate parameter (of no importance here), and the mean of this distribution is therefore $\mu.$ We use the log-link

$$\log\left( \mu\right)=\eta$$

where $\eta$ is the linear predictor (i.e., $\mu=e^{\eta}$), which ensures that $\mu>0$.

The only new parameter introduced here is $\phi\sim Cauchy\left( 0,2.5 \right)$ (the prior, truncated at 0).

### balancemean

The histograms for this variable in Multimedia Appendix 1, Figure S2, indicate a J-shaped distribution, which cannot be modeled by a Gamma nor a skew normal distribution. In order to model this, we switch to a Beta distribution, which is highly flexible with shape depending on the parameters, and allowing for the form shown in the Figure.

If

$$y\sim Beta\left( a,b \right),$$

$$a>0, b>0$$

then the mean of $y$ is:

$$\frac{a}{a+b}$$

Hence, we take the likelihood as

$$y\sim Beta\left( \phi\theta,\phi\left( 1-\theta\right) \right)$$

since then the mean of $y$ is $\theta$, and we use the logistic link:

$$\theta= \frac{1}{1+e^{-\eta}}$$

where $\eta$ is the linear predictor from Equation (1). $\phi>0$ is a scale parameter which can be estimated from the data, and is not of interest here. Its prior distribution is

$\phi\sim Gamma\left( shape=2,rate=0.1 \right)$.

This gives the connection between the linear predictor and the mean of the distribution and also ensures that $\theta$ is in the range [0,1] as required.

### tmtbmistakes

This is a count variable recording the number of events (mistakes) that occur over a time period. This is typically modelled by a Poisson distribution, which has the property that the mean and variance of the distribution are equal. For the Current Self condition the mean (variance) values are Current Self: 0.73(1.160) and for Young Self: 0.32(0.407) so that at least in the Current Age condition the requirement is not met. A more general alternative is to use the Negative Binomial distribution. This can be formulated with 2 parameters, the mean $\mu$ and $\phi$, where the variance is: $\mu+\frac{\mu^{2}}{\phi}$. This is the formulation used by the Stan software^[[1]](#footnote-1)^. For large $\phi$ the Negative Binomial tends to the Poisson. Hence we used the Negative Binomial distribution. The prior distribution is:

$$\phi\sim Gamma\left( shape=2,rate=0.1 \right)$$

## Prior 95% HDIs

Table S2 shows the prior 95% HDIs for all of the prior distributions that we have used. All of the intervals are wide. These are ‘weakly informative’ prior distributions [3]. In interpreting the results it is important to examine the posterior HDIs in order to check how much the observed data has narrowed down these intervals.

**Table S2**. The 95% prior highest density intervals for the distributions used as priors

|  | **95% HDI** | |
| --- | --- | --- |
| **Distribution** | **Lower bound** | **Upper Bound** |
| $normal\left( mean0,sigma=2.5 \right)$ | -4.90 | 4.90 |
| $normal\left( location=0,sigma=2.5 \right)$, lower bound=0 | 0.0 | 4.90 |
| $Gamma\left( shape=2,rate=0.1 \right)$ | 0.42 | 47.65 |

## References

[1] Azzalini, A., 1985. A class of distributions which includes the normal ones. *Scandinavian journal of statistics*, 171-178.

[2] O'hagan, A. and Leonard, T., 1976. Bayes estimation subject to uncertainty about parameter constraints. *Biometrika 63*, 1, 201-203. <http://dx.doi.org/10.1093/biomet/63.1.201>.

[3] Lemoine, N.P., 2019. Moving beyond noninformative priors: why and how to choose weakly informative priors in Bayesian analyses. *Oikos 128*, 7, 912-928. <http://dx.doi.org/10.1111/oik.05985>.

1. <https://mc-stan.org/docs/2_19/functions-reference/nbalt.html#nbalt> [↑](#footnote-ref-1)
