# Supplementary material for: Counterclockwise Virtual Reality–Based Embodiment of a Younger Self and Revisit of a Past Iconic Event in Older Adults: Between-Groups Study of Cognitive and Physical Performance
Source: JMIR Form Res. 2026 Apr 22;10:e88338. doi: 10.2196/88338 (PMC13102333; doi:10.2196/88338)
Supplement: Multimedia Appendix 5 [file formative-v10-e88338-s005.docx]

**Table S1**. Summaries of the posterior distributions of the parameters of the subjective variables of Table 3, showing the mean, SD, 95% HDI and the probability of the parameter being positive.

| Parameter | **Interpretation** | Mean | SD | 2.5% | 97.5% | P(>0) |
| --- | --- | --- | --- | --- | --- | --- |
| **subjectiveage** |  |  |  |  |  |  |
| $\mu_{sage}$ |  | -1.367 | 0.287 | -1.924 | -0.790 | 0.000 |
| $\alpha_{sage,2}$ | Session | 0.627 | 0.306 | 0.017 | 1.221 | 0.979 |
| $\alpha_{sage,3}$ | Main | 0.431 | 0.323 | -0.217 | 1.052 | 0.908 |
| $\alpha_{sage,4}$ | Effects | 0.706 | 0.306 | 0.116 | 1.313 | 0.988 |
| $\alpha_{sage,5}$ |  | 0.562 | 0.307 | -0.030 | 1.180 | 0.965 |
| $\beta_{sage,2}$ | Condition Session 1 | 0.684 | 0.430 | -0.147 | 1.540 | 0.943 |
| $\gamma_{sage,2,2}$ | Interaction effects | -0.543 | 0.421 | -1.369 | 0.278 | 0.098 |
| $\gamma_{sage,2,3}$ | *Condition (Young)* | -0.508 | 0.432 | -1.338 | 0.361 | 0.121 |
| $\gamma_{sage,2,4}$ | *×* | -0.725 | 0.428 | -1.539 | 0.137 | 0.046 |
| $\gamma_{sage,2,5}$ | *Session* | -0.314 | 0.432 | -1.145 | 0.540 | 0.233 |
| $\kappa_{sage,1}$ | Shape | 0.578 | 1.462 | -2.153 | 3.390 | 0.636 |
| $\kappa_{sage,2}$ |  | -0.168 | 2.440 | -4.580 | 4.472 | 0.460 |
| **philadelphiatotal** |  |  |  |  |  |  |
| $\mu_{phil}$ |  | -0.095 | 0.244 | -0.576 | 0.378 | 0.349 |
| $\alpha_{phil,2}$ | Session | 0.217 | 0.251 | -0.285 | 0.689 | 0.804 |
| $\alpha_{phil,3}$ | Main | 0.258 | 0.249 | -0.234 | 0.737 | 0.849 |
| $\alpha_{phil,4}$ | Effects | 0.109 | 0.249 | -0.388 | 0.590 | 0.670 |
| $\alpha_{phil,5}$ |  | 0.208 | 0.240 | -0.264 | 0.682 | 0.808 |
| $\beta_{phil,2}$ | Condition Session 1 | 0.269 | 0.416 | -0.554 | 1.084 | 0.743 |
| $\gamma_{phil,2,2}$ | Interaction effects | -0.376 | 0.344 | -1.041 | 0.310 | 0.135 |
| $\gamma_{phil,2,3}$ | *Condition (Young)* | -0.310 | 0.348 | -0.985 | 0.371 | 0.185 |
| $\gamma_{phil,2,4}$ | *×* | -0.126 | 0.340 | -0.776 | 0.552 | 0.353 |
| $\gamma_{phil,2,5}$ | *Session* | -0.257 | 0.328 | -0.910 | 0.369 | 0.216 |
| $\kappa_{phil,1}$ | Shape | 0.644 | 1.919 | -3.024 | 4.224 | 0.631 |
| $\kappa_{phil,2}$ |  | -1.666 | 2.181 | -5.783 | 2.534 | 0.231 |
| **AARC Negative** |  |  |  |  |  |  |
| $\mu_{aarcn}$ |  | -0.026 | 0.308 | -0.633 | 0.568 | 0.475 |
| $\alpha_{aarcn,2}$ | Session | 0.079 | 0.300 | -0.501 | 0.668 | 0.601 |
| $\alpha_{aarcn,3}$ | Main | -0.507 | 0.318 | -1.130 | 0.116 | 0.058 |
| $\alpha_{aarcn,4}$ | Effects | -0.466 | 0.309 | -1.079 | 0.133 | 0.065 |
| $\alpha_{aarcn,5}$ |  | -0.342 | 0.323 | -0.962 | 0.300 | 0.146 |
| $\beta_{aarcn,2}$ | Condition Session 1 | 0.413 | 0.440 | -0.478 | 1.265 | 0.827 |
| $\gamma_{aarcn,2,2}$ | Interaction effects | -0.366 | 0.394 | -1.123 | 0.419 | 0.176 |
| $\gamma_{aarcn,2,3}$ | *Condition (Young)* | 0.108 | 0.404 | -0.701 | 0.890 | 0.610 |
| $\gamma_{aarcn,2,4}$ | *×* | 0.063 | 0.401 | -0.720 | 0.847 | 0.561 |
| $\gamma_{aarcn,2,5}$ | *Session* | -0.302 | 0.414 | -1.137 | 0.487 | 0.231 |
| $\kappa_{aarcn,1}$ | Shape | -0.621 | 1.416 | -3.388 | 2.083 | 0.349 |
| $\kappa_{aarcn,2}$ |  | 0.011 | 2.627 | -4.594 | 4.803 | 0.502 |
| **AARC Positive** |  |  |  |  |  |  |
| $\mu_{aarcp}$ |  | -0.128 | 0.316 | -0.760 | 0.483 | 0.343 |
| $\alpha_{aarcp,2}$ | Session | 0.315 | 0.388 | -0.456 | 1.074 | 0.795 |
| $\alpha_{aarcp,3}$ | Main | 0.099 | 0.407 | -0.690 | 0.908 | 0.589 |
| $\alpha_{aarcp,4}$ | Effects | -0.469 | 0.383 | -1.217 | 0.282 | 0.109 |
| $\alpha_{aarcp,5}$ |  | -0.223 | 0.395 | -1.017 | 0.523 | 0.281 |
| $\beta_{aarcp,2}$ | Condition Session 1 | 0.193 | 0.400 | -0.575 | 0.995 | 0.685 |
| $\gamma_{aarcp,2,2}$ | Interaction effects | -0.239 | 0.478 | -1.189 | 0.678 | 0.311 |
| $\gamma_{aarcp,2,3}$ | *Condition (Young)* | -0.099 | 0.502 | -1.115 | 0.842 | 0.426 |
| $\gamma_{aarcp,2,4}$ | *×* | 0.587 | 0.479 | -0.351 | 1.524 | 0.891 |
| $\gamma_{aarcp,2,5}$ | *Session* | 0.442 | 0.482 | -0.486 | 1.394 | 0.818 |
| $\kappa_{aarcp,1}$ | Shape | -1.071 | 1.073 | -3.066 | 0.891 | 0.183 |
| $\kappa_{aarcp,2}$ |  | 3.422 | 1.823 | 0.542 | 8.525 | 0.953 |
| **wellbeingscale** |  |  |  |  |  |  |
| $\mu_{well}$ |  | 0.618 | 0.234 | 0.143 | 1.064 | 0.995 |
| $\alpha_{well,2}$ | Session | -0.108 | 0.200 | -0.504 | 0.284 | 0.293 |
| $\alpha_{well,3}$ | Main | 0.099 | 0.208 | -0.310 | 0.503 | 0.685 |
| $\alpha_{well,4}$ | Effects | 0.085 | 0.200 | -0.308 | 0.479 | 0.667 |
| $\alpha_{well,5}$ |  | -0.027 | 0.199 | -0.418 | 0.367 | 0.443 |
| $\beta_{well,2}$ | Condition Session 1 | -0.744 | 0.412 | -1.561 | 0.043 | 0.033 |
| $\gamma_{well,2,2}$ | Interaction effects | 0.271 | 0.257 | -0.237 | 0.772 | 0.855 |
| $\gamma_{well,2,3}$ | *Condition (Young)* | 0.162 | 0.265 | -0.343 | 0.696 | 0.730 |
| $\gamma_{well,2,4}$ | *×* | 0.261 | 0.26 | -0.259 | 0.761 | 0.844 |
| $\gamma_{well,2,5}$ | *Session* | 0.063 | 0.261 | -0.461 | 0.566 | 0.598 |
| $\kappa_{well,1}$ | Shape | -0.579 | 1.321 | -3.018 | 1.872 | 0.343 |
| $\kappa_{well,2}$ |  | 2.970 | 1.673 | -1.097 | 6.286 | 0.951 |

**Table S2**. Summaries of the posterior distributions of the parameters of the performance variables of Table 3, showing the mean, SD, 95% HDI and the probability of the parameter being positive.

| **Parameter** | **Interpretation** | **Mean** | **SD** | **2.5%** | **97.5%** | **P(>0)** |
| --- | --- | --- | --- | --- | --- | --- |
| **TMTAtime** |  |  |  |  |  |  |
| $\mu_{tmtat}$ |  | 3.680 | 0.068 | 3.548 | 3.816 | 1.000 |
| $\alpha_{tmtat,2}$ | Session | 0.023 | 0.077 | -0.124 | 0.175 | 0.618 |
| $\alpha_{tmtat,3}$ | Main | -0.152 | 0.083 | -0.319 | 0.004 | 0.033 |
| $\alpha_{tmtat,4}$ | Effects | 0.037 | 0.076 | -0.113 | 0.183 | 0.688 |
| $\alpha_{tmtat,5}$ |  | -0.281 | 0.089 | -0.451 | -0.106 | 0.001 |
| $\beta_{tmtat,2}$ | Condition Session 1 | 0.086 | 0.100 | -0.106 | 0.288 | 0.812 |
| $\gamma_{tmtat,2,2}$ | Interaction effects | -0.185 | 0.112 | -0.403 | 0.038 | 0.048 |
| $\gamma_{tmtat,2,3}$ | *Condition (Young)* | -0.132 | 0.117 | -0.359 | 0.099 | 0.130 |
| $\gamma_{tmtat,2,4}$ | *×* | -0.417 | 0.114 | -0.641 | -0.193 | 0.000 |
| $\gamma_{tmtat,2,5}$ | *Session* | -0.011 | 0.122 | -0.25 | 0.228 | 0.466 |
| $\phi_{tmtat}$ | Rate | 0.765 | 0.122 | 0.53 | 1.004 | 1.000 |
| **TMTBtime** |  |  |  |  |  |  |
| $\mu_{tmtbt}$ |  | 4.59 | 0.09 | 4.406 | 4.761 | 1.000 |
| $\alpha_{tmtbt,2}$ | Session | -0.094 | 0.081 | -0.257 | 0.059 | 0.121 |
| $\alpha_{tmtbt,3}$ | Main | -0.189 | 0.085 | -0.353 | -0.017 | 0.014 |
| $\alpha_{tmtbt,4}$ | Effects | -0.222 | 0.083 | -0.385 | -0.057 | 0.005 |
| $\alpha_{tmtbt,5}$ |  | -0.196 | 0.089 | -0.377 | -0.027 | 0.015 |
| $\beta_{tmtbt,2}$ | Condition Session 1 | -0.156 | 0.127 | -0.407 | 0.089 | 0.102 |
| $\gamma_{tmtbt,2,2}$ | Interaction effects | 0.087 | 0.118 | -0.136 | 0.328 | 0.775 |
| $\gamma_{tmtbt,2,3}$ | *Condition (Young)* | 0.028 | 0.12 | -0.209 | 0.264 | 0.593 |
| $\gamma_{tmtbt,2,4}$ | *×* | -0.099 | 0.121 | -0.333 | 0.142 | 0.208 |
| $\gamma_{tmtbt,2,5}$ | *Session* | -0.043 | 0.124 | -0.293 | 0.197 | 0.364 |
| $\phi_{tmtbt}$ | Rate | 0.328 | 0.055 | 0.227 | 0.441 | 1.000 |
| **TMTBmistakes** |  |  |  |  |  |  |
| $\mu_{tmtbm}$ |  | -0.493 | 0.423 | -1.314 | 0.339 | 0.116 |
| $\alpha_{tmtbm,2}$ | Session | 0.472 | 0.521 | -0.568 | 1.493 | 0.825 |
| $\alpha_{tmtbm,3}$ | Main | 0.142 | 0.560 | -1.007 | 1.206 | 0.604 |
| $\alpha_{tmtbm,4}$ | Effects | -0.272 | 0.596 | -1.446 | 0.886 | 0.329 |
| $\alpha_{tmtbm,5}$ |  | 0.39 | 0.560 | -0.711 | 1.492 | 0.760 |
| $\beta_{tmtbm,2}$ | Condition Session 1 | -0.468 | 0.633 | -1.73 | 0.738 | 0.232 |
| $\gamma_{tmtbm,2,2}$ | Interaction effects | -0.856 | 0.892 | -2.602 | 0.9 | 0.166 |
| $\gamma_{tmtbm,2,3}$ | *Condition (Young)* | -0.126 | 0.841 | -1.757 | 1.536 | 0.439 |
| $\gamma_{tmtbm,2,4}$ | *×* | -1.402 | 1.158 | -3.723 | 0.81 | 0.108 |
| $\gamma_{tmtbm,2,5}$ | *Session* | -0.889 | 0.915 | -2.664 | 0.934 | 0.165 |
| $\phi_{tmtbm}$ |  | 8.762 | 10.989 | 0.237 | 31.427 | 1.000 |
| **gripstrengthRmean** |  |  |  |  |  |  |
| $\mu_{gripr}$ |  | 3.633 | 0.048 | 3.54 | 3.728 | 1.000 |
| $\alpha_{gripr,2}$ | Session | 0.023 | 0.032 | -0.043 | 0.084 | 0.762 |
| $\alpha_{gripr,3}$ | Main | 0.055 | 0.033 | -0.01 | 0.118 | 0.953 |
| $\alpha_{gripr,4}$ | Effects | 0.056 | 0.031 | -0.006 | 0.118 | 0.962 |
| $\alpha_{gripr,5}$ |  | 0.075 | 0.032 | 0.013 | 0.138 | 0.990 |
| $\beta_{gripr,2}$ | Condition Session 1 | 0.393 | 0.158 | 0.076 | 0.701 | 0.994 |
| $\gamma_{gripr,2,2}$ | Interaction effects | 0.005 | 0.047 | -0.087 | 0.097 | 0.542 |
| $\gamma_{gripr,2,3}$ | *Condition (Young)* | -0.012 | 0.047 | -0.103 | 0.081 | 0.395 |
| $\gamma_{gripr,2,4}$ | *×* | 0.047 | 0.046 | -0.042 | 0.137 | 0.849 |
| $\gamma_{gripr,2,5}$ | *Session* | 0.022 | 0.046 | -0.068 | 0.113 | 0.682 |
| $\phi_{gripr}$ | Rate | 2.815 | 0.443 | 1.968 | 3.693 | 1.000 |
| **gripstrengthLmean** |  |  |  |  |  |  |
| $\mu_{gripl}$ |  | 3.799 | 0.04 | 3.72 | 3.879 | 1.000 |
| $\alpha_{gripl,2}$ | Session | -0.009 | 0.031 | -0.069 | 0.052 | 0.381 |
| $\alpha_{gripl,3}$ | Main | -0.006 | 0.031 | -0.067 | 0.055 | 0.417 |
| $\alpha_{gripl,4}$ | Effects | 0.003 | 0.03 | -0.057 | 0.062 | 0.544 |
| $\alpha_{gripl,5}$ |  | 0.007 | 0.03 | -0.052 | 0.068 | 0.59 |
| $\beta_{gripl,2}$ | Condition Session 1 | 0.165 | 0.112 | -0.058 | 0.386 | 0.931 |
| $\gamma_{gripl,2,2}$ | Interaction effects | 0.073 | 0.045 | -0.015 | 0.162 | 0.949 |
| $\gamma_{gripl,2,3}$ | *Condition (Young)* | 0.096 | 0.045 | 0.007 | 0.184 | 0.983 |
| $\gamma_{gripl,2,4}$ | *×* | 0.119 | 0.044 | 0.033 | 0.206 | 0.996 |
| $\gamma_{gripl,2,5}$ | *Session* | 0.12 | 0.044 | 0.034 | 0.208 | 0.996 |
| $\phi_{gripl}$ | Rate | 3.369 | 0.536 | 2.345 | 4.409 | 1.000 |
| **balancemean** |  |  |  |  |  |  |
| $\mu_{blnc}$ |  | 2.561 | 0.343 | 1.913 | 3.251 | 1.000 |
| $\alpha_{blnc,2}$ | Session | 0.127 | 0.422 | -0.712 | 0.947 | 0.619 |
| $\alpha_{blnc,3}$ | Main | 0.043 | 0.432 | -0.805 | 0.888 | 0.536 |
| $\alpha_{blnc,4}$ | Effects | 0.049 | 0.421 | -0.764 | 0.878 | 0.548 |
| $\alpha_{blnc,5}$ |  | 0.093 | 0.424 | -0.702 | 0.96 | 0.586 |
| $\beta_{blnc,2}$ | Condition Session 1 | -0.396 | 0.453 | -1.269 | 0.508 | 0.189 |
| $\gamma_{blnc,2,2}$ | Interaction effects | 0.146 | 0.608 | -1.061 | 1.331 | 0.596 |
| $\gamma_{blnc,2,3}$ | *Condition (Young)* | 0.206 | 0.627 | -1.037 | 1.426 | 0.632 |
| $\gamma_{blnc,2,4}$ | *×* | -0.038 | 0.606 | -1.198 | 1.171 | 0.477 |
| $\gamma_{blnc,2,5}$ | *Session* | 0.101 | 0.619 | -1.11 | 1.315 | 0.565 |
| $\phi_{blnc}$ | Scale | 2.886 | 0.533 | 1.903 | 3.954 | 1.000 |
| **walktime** |  |  |  |  |  |  |
| $\mu_{wlkt}$ |  | 1.578 | 0.069 | 1.445 | 1.715 | 1.000 |
| $\alpha_{wlkt,2}$ | Session | -0.054 | 0.089 | -0.231 | 0.113 | 0.275 |
| $\alpha_{wlkt,3}$ | Main | -0.044 | 0.087 | -0.211 | 0.129 | 0.308 |
| $\alpha_{wlkt,4}$ | Effects | -0.082 | 0.088 | -0.253 | 0.091 | 0.178 |
| $\alpha_{wlkt,5}$ |  | -0.066 | 0.088 | -0.24 | 0.106 | 0.23 |
| $\beta_{wlkt,2}$ | Condition Session 1 | 0.048 | 0.100 | -0.146 | 0.245 | 0.687 |
| $\gamma_{wlkt,2,2}$ | Interaction effects | 0.097 | 0.127 | -0.141 | 0.356 | 0.777 |
| $\gamma_{wlkt,2,3}$ | *Condition (Young)* | 0.034 | 0.125 | -0.218 | 0.28 | 0.611 |
| $\gamma_{wlkt,2,4}$ | *×* | 0.064 | 0.125 | -0.186 | 0.302 | 0.696 |
| $\gamma_{wlkt,2,5}$ | *Session* | -0.042 | 0.131 | -0.298 | 0.216 | 0.375 |
| $\phi_{wlkt}$ | Rate | 4.758 | 0.602 | 3.599 | 5.945 | 1.000 |
